# MiRNA Dysregulation in Epilepsy: Bridging Molecular Mechanisms and Therapeutic Innovation

**DOI:** 10.1007/s12035-026-05926-5

**Published:** 2026-06-26

**Authors:** Reda M. Mansour, Hend H. Mohamed, Khaled M. Alam-Eldein, Mohamed Hemdan, Nehal I. Rizk, Osama A. Mohammed, Youssef A. Doghish, Mariam O. Abbass, Moaz Mohsen Shafey, Moustafa Mahmoud Abdelaziz, Mohamed H. A. Gadelmawla, Amal Ahmed Mohamed, Ahmed S. Doghish

**Affiliations:** 1 Zoology and Entomology Department, Faculty of Science, Helwan University, Helwan, Egypt; 2 Molecular Biology and Biotechnology Department, School of Biotechnology, Badr University in Cairo (BUC), Badr City, Cairo, 11829 Egypt; 3 School of Biotechnology, Badr University in Cairo (BUC), Badr City, Cairo, 11829 Egypt; 4 Department of Biochemistry, Faculty of Pharmacy and Drug Technology, Egyptian Chinese University, Cairo, 11786 Egypt; 5 Department of Pharmacology, College of Medicine, University of Bisha, 61922 Bisha, Saudi Arabia; 6 Faculty of Dentistry, Badr University in Cairo (BUC), Badr City, Cairo, 11829 Egypt; 7 Faculty of Medicine, Ain Shams University, Cairo, 11591 Egypt; 8 Life Sciences Department, Faculty of Biotechnology, Sinai University, Ismailia, 41636 Egypt; 9 Department of Biochemistry and Molecular Biology, National Hepatology and Tropical Medicine Research Institute, GOTHI, Cairo, Egypt; 10 Biochemistry and Molecular Biology Department, Faculty of Pharmacy (Boys), Al-Azhar University, Nasr CityCairo, 11231 Egypt; 11 Department of Biochemistry, Faculty of Pharmacy, Badr University in Cairo (BUC), Badr CityCairo, 11829 Egypt

**Keywords:** MicroRNAs, Epilepsy, Neuroinflammation, Synaptic remodeling, Therapeutic target, Diagnosis, Prognosis

## Abstract

MicroRNAs (miRNAs), small non-coding RNA molecules, have been considered as pivotal regulators of gene expression, influencing many biological functions, including nerve cell development, synaptic plasticity, and inflammatory responses. Their biogenesis involves a multi-step process, beginning with transcription by RNA polymerase II, followed by processing through Drosha and Dicer enzymes, culminating in the formation of mature miRNAs that integrate into the RNA-induced silencing complex [1] to control target messenger RNA (mRNA) stability and translation. In the context of epilepsy, a neurological disorder characterized by recurrent seizures, miRNAs have a role in the modulation of neuronal excitability and network synchronization. Dysregulation of specific miRNAs can disrupt the delicate balance between excitatory and inhibitory neurotransmission, contributing to the pathogenesis of epilepsy. Moreover, miRNAs influence neuroplasticity by regulating genes involved in synaptic remodeling and neuronal connectivity, processes that are often aberrant in epileptic brains. Inflammatory pathways are also modulated by miRNAs, with certain miRNAs acting as key regulators of cytokine expression and immune cell activation, thereby influencing neuroinflammation associated with epileptogenesis. Clinically, altered miRNA expression profiles have been shown in the blood and cerebrospinal fluid of epilepsy patients, suggesting their potential as non-invasive biomarkers for diagnosis and prognosis. Furthermore, therapeutic strategies targeting miRNAs are being explored, aiming to restore normal gene expression patterns and mitigate seizure activity. This review delves into the intricate roles of miRNAs in epilepsy, encompassing their biogenesis, involvement in disease pathophysiology, impact on neuroplasticity and inflammation, and their emerging clinical applications.

**Graphical Abstract:**

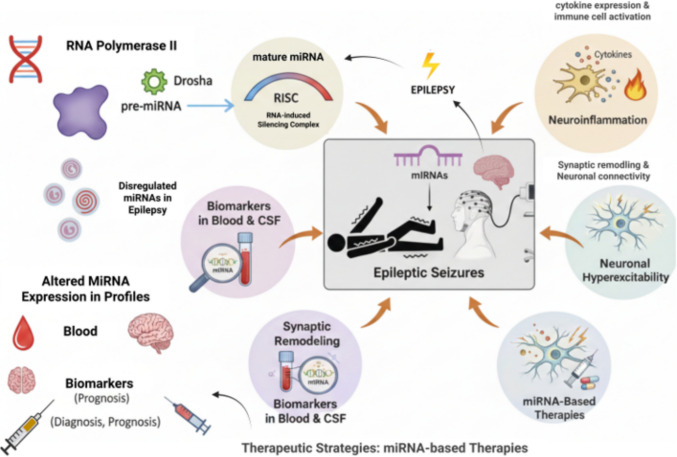

## Introduction

Epilepsy, a prevalent neurological disorder, affects approximately 70 million individuals worldwide and is characterized by recurrent, unprovoked seizures resulting from abnormal neuronal activity [2]. Despite significant advances in clinical diagnosis and management, the molecular mechanisms underlying epilepsy remain incompletely understood. Increasing attention has been directed toward post-transcriptional regulatory mechanisms as key contributors to neuronal dysfunction and disease progression. [3].

**Fig. 1:**
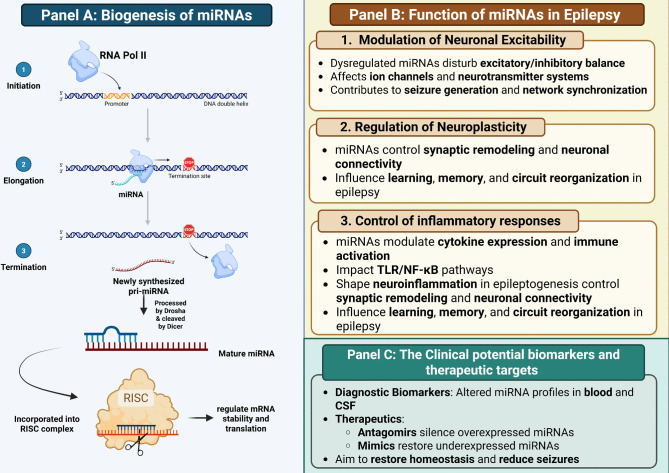
Left panel (Panel **A**) illustrates miRNA biogenesis, from transcription by RNA polymerase II through to the elongation phase, where the pre-miRNA is synthesized. The right panel (Panel **B**) shows the functional roles of miRNAs in epilepsy, including their modulation of neuronal excitability, synaptic plasticity, and neuroinflammation. These processes contribute to epilepsy, with miRNAs also serving as potential biomarkers and therapeutic targets, which are shown in Panel **C**

MiRNAs are formed of 22-nucleotide-long non-coding RNAs that control gene expression post-transcriptionally. Their biogenesis is modulated by the transcription of primary miRNA (pri-miRNA) transcripts by RNA polymerase II or III (Fig. 1), followed by nuclear processing by the Drosha–DGCR8 complex to generate precursor miRNAs (pre-miRNAs). These pre-miRNAs are subsequently exported to the cytoplasm via Exportin-5, where Dicer further processes them into mature miRNA duplexes. One strand of the duplex is incorporated into the RNA-induced silencing complex (RISC), enabling sequence-specific repression of target messenger RNAs through translational inhibition or mRNA degradation [1, 4–6]. In addition to intracellular regulation, miRNAs can be secreted into extracellular fluids and migrate to target cells via vesicles, such as exosomes, or by conjugating with proteins, acting as chemical messengers to mediate cell–cell communication [7].

Epilepsy's pathophysiology involves a complex interplay of genetic, molecular, and cellular factors leading to neuronal hyperexcitability and hyper-synchronization [8]. Key mechanisms include an imbalance between excitatory and inhibitory neurotransmission, alterations in ion channel function, and synaptic reorganization. Neuroinflammation, characterized by the activation of glial cells and the release of pro-inflammatory cytokines, also plays a crucial role in epileptogenesis. Additionally, blood–brain barrier (BBB) disruption and oxidative stress contribute to the chronicity and severity of the disorder [5, 9].

MiRNAs have emerged as critical regulators in the pathogenesis of epilepsy. They influence various pathogenic processes, including inflammatory responses, neuronal necrosis and apoptosis, dendritic growth, and synaptic remodeling. These findings underscore the significance of miRNA dysregulation in epilepsy's molecular landscape [10].

Neuroplasticity, the brain's ability to reorganize its structure and function, is a fundamental process in both normal and pathological conditions. In epilepsy, aberrant neuroplastic changes, such as mossy fiber sprouting and altered dendritic spine density, contribute to the development and maintenance of hyperexcitable neuronal networks. miRNAs are instrumental in regulating genes involved in synaptic plasticity [11, 12]. For example, miR-132 modulates the expression of proteins critical for dendritic growth and spine formation, and its dysregulation has been linked to epileptogenesis. Thus, miRNAs serve as key modulators of the neuroplastic alterations observed in epilepsy [7, 13].

Inflammation is a hallmark of epilepsy, contributing to neuronal hyperexcitability and seizure propagation. miRNAs regulate the expression of various inflammatory cytokines and receptors. For instance, mouse miR-155 promotes the production of pro-inflammatory cytokines [14], while miR-146a acts as a negative feedback regulator to dampen excessive inflammatory responses. The delicate balance maintained by these miRNAs is crucial for modulating the neuroinflammatory milieu in epilepsy. Disruption of this balance can exacerbate inflammation, leading to increased seizure susceptibility and severity[15].

The stability and specificity of miRNAs in biological fluids make them attractive candidates for non-invasive biomarkers in epilepsy. Circulating miRNAs, such as miR-134 and miR-146a, have been proposed in epileptic human patients as potential diagnostic and prognostic markers [16]. Elevated levels of miR-134 in plasma have been associated with drug-resistant epilepsy, suggesting its utility in predicting treatment response. Furthermore, miRNA expression profiles can aid in distinguishing between different epilepsy subtypes and in monitoring disease progression. The development of miRNA-based diagnostic tools holds promise for personalized medicine approaches in epilepsy management [17].

Their dysregulation contributes to the pathogenesis of epilepsy, and their stability in biological fluids positions them as promising biomarkers for diagnosis and prognosis[2].

To facilitate reference and verification, the mature sequences of the discussed miRNAs were retrieved and listed in Table 1, with direct database links provided from miRBase. Understanding the intricate network of miRNA-mediated regulation offers new avenues for therapeutic intervention and underscores the potential of miRNAs in advancing epilepsy research and clinical practice [18].

**Table 1:** Bioinformatics databases and online resources used in this review for comprehensive miRNA analysis, including sequence verification, target prediction, and disease association

Link	Purpose	Description	Database
https://www.mirbase.org	Cite exact sequences and accessions, check aliases	Canonical repository of miRNA hairpin/mature sequences with MI/MIMAT accessions and nomenclature	miRBase
https://www.mirgenedb.org	Consistent naming across species evolutionary context	Expert-curated, gene-level miRNA families and orthology across many species	MirGeneDB
https://rnacentral.org	Use a unified ncRNA ID to access external resources	Aggregates non-coding RNA data (incl. miRNAs) and cross-links to source databases	RNAcentral
https://www.targetscan.org	Build hypotheses on likely mRNA targets	Conserved seed-based target predictions with context + + scores	TargetScan (Human/Mouse)
http://mirdb.org	Alternative predictions independent of conservation	ML-based target predictions (MirTarget) from large RNA-seq datasets	miRDB
https://mirtarbase.cuhk.edu.cn	Find validated targets with evidence type	Manually curated, experimentally validated miRNA target interactions (MTIs)	miRTarBase
https://rnasysu.com/encori/	Prioritize interactions with AGO-CLIP support	CLIP-supported miRNA RNA interaction maps; ceRNA networks	starBase/ENCORI
http://www.cuilab.cn/hmdd	Link dysregulated miRNAs to disease evidence	Curated associations between human miRNAs and diseases	HMDD (Human miRNA Disease DB)
https://dianalab.e-ce.uth.gr/mited	Compare expression across many studies/tissues	Uniformly processed small-RNA datasets with miRNA/isomiR expression	DIANA miTED
http://www.exocarta.org	Context for miRNAs detected in exosomes	Community-curated exosome cargo (RNAs/proteins/lipids)	ExoCarta
http://microvesicles.org	Cross-reference EV miRNA evidence and sources	Broad EV cargo compendium across organisms and fluids	Vesiclepedia

miRNAs: micro RNAs, ncRNA: non-coding RNAs, mRNA: messenger RNAs, MTIs: miRNA target interactions, EV: extracellular vesicles

## Molecular Pathophysiology of Epilepsy

Epileptogenesis involves complex molecular processes with key contributing factors including an imbalance between excitatory and inhibitory neurotransmission, abnormal synaptic plasticity, excessive neural network activity, inflammation, and immune dysfunction (Table 2). This study primarily explores how these mechanisms contribute to the onset of epilepsy [19].

**Table 2:** Molecular pathophysiology of epilepsy

Category	Factor/Mechanism	Description	Ref
i) Blood–brain barrier (BBB) dysfunction	BBB & IgG leakage	Leakage of IgG may contribute to neuronal impairment in treatment-resistant epilepsy, particularly when there is BBB disruption of autoimmune origin	[25]
	P-glycoprotein overexpression	Increased expression of efflux transporters such as P-glycoprotein at the BBB limits drug penetration and contributes to pharmacoresistance	[25, 31]
ii) Structure and/or functionality of target ion channels and neurotransmitters	Excitatory/inhibitory imbalance	Decreased inhibitory (GABAergic) or increased excitatory (glutamatergic) signaling disturbs network stability and promotes seizures	[21]
	Ion-gated channels	Ion-channel gene function can be altered by genetic mutations, infections, or immune responses (e.g., antibodies, CTLs), resulting in abnormal neuronal firing	[22, 32]
iii) Structural brain alterations and/or network changes	Synaptic plasticity	Rapid modifications in synaptic strength and inhibitory synapse function promote hyperexcitability and the initiation of seizures	[22, 32, 33]
	γ and β frequency modulation	Dynamic modulation of hippocampal γ and β rhythms enhances synaptic connectivity and contributes to abnormal synchronization	[24]
iv) Genetic variants of proteins	Synapsins	Alterations or deletions in synapsin genes disrupt the equilibrium between excitatory and inhibitory neurotransmission, predisposing to epilepsy	[21]
v) Inflammation and immune mechanisms	Inflammatory signaling	Inflammatory responses disturb neural transmission and homeostasis, exacerbating seizure activity	[26, 27]
	IL-6 and TNF-α	Elevated levels are strongly associated with seizure occurrence and severity	[24, 27]
	Encephalitis and COX-2	Inflammatory induction of COX-2 during encephalitis and prolonged seizures contributes to neuronal injury	[29, 30]
vi) Increased intrinsic disease severity	Persistent hyperexcitability	Chronic impairment of synaptic plasticity and excitatory–inhibitory imbalance maintains heightened seizure susceptibility and poor treatment response	[22, 32, 33]

BBB: blood–brain barrier, IgG: immunoglobulin G, GABA: gamma-aminobutyric acid, CTLs: cytotoxic T lymphocytes, IL-6: interleukin 6, TNF-α: tumor necrosis factor α, COX-2: cyclooxygenase 2

### Neurotransmitter Imbalance

Epileptic seizures often result from chronic imbalances in brain signaling. A common mechanism involves either a decrease in inhibitory neurotransmitters like gamma-aminobutyric acid [20] or an increase in excitatory neurotransmitters such as glutamate. Genetic mutations—such as those affecting synapsin genes—can disrupt this balance, promoting hyperexcitability. For example, mutations in the stargazin gene and the LGI1/ADAM22 complex, which are involved in regulating AMPA receptor function, have been linked to seizure activity [21].

### Abnormal Synaptic Plasticity and Hyperexcitability

Abnormal changes in ion channel function also play a role. Ion channels regulate how ions move across neuron membranes, and their function can be disrupted by genetic mutations, pathogens, or immune responses. For instance, CD8 + T cells releasing cytotoxic granules may interfere with ion channel operation, altering neuron excitability. Moreover, synaptic plasticity, how synapse strength changes over time, can become dysfunctional, leading to seizure generation. Both excitatory and inhibitory synapses may be involved. Pro-inflammatory cytokines such as interleukin-6 (IL-6) and macrophage inflammatory proteins (MIP), produced by astrocytes and microglia, further enhance neural excitability [21–23]. Studies suggest that immune signaling molecules can influence both synaptic transmission and plasticity. Rapid changes in synaptic strength during γ and β frequency activity in the hippocampus may contribute directly to seizure initiation [24, 25].

### Inflammation and Immune Dysregulation in Epilepsy

Inflammatory cells release signaling molecules that disrupt normal neuronal activity, potentially leading to seizures. In mouse models lacking synapsin 2, elevated levels of pro-inflammatory cytokines such as IL-6 and tumor necrosis factor (TNF) have been observed, alongside increased brain inflammation and altered neurogenesis in the hippocampus [26, 27]. Additionally, central nervous system (CNS) inflammation triggered by BBB breakdown is closely linked to both the initiation and progression of epilepsy. This is evidenced by the presence of immunoglobulin G (IgG) deposits in brain regions where seizures originate, indicating BBB dysfunction [25, 28]. The immune system's involvement is further supported by the detection of IgGs in the brain during seizure conditions, suggesting an autoimmune component in epileptic pathogenesis [25]. Moreover, conditions like encephalitis may worsen the situation, as inflammation-induced overexpression of cyclooxygenase-2 (COX-2) during prolonged seizures/Status epilepticus (SE) contributes to neuronal death [29, 30].

## Role of miRNAs in Epilepsy Pathogenesis

### miRNAs as Key Regulators in Epilepsy Pathogenesis

miRNAs play a pivotal role in regulating key molecular processes involved in epilepsy. For instance, miR-146a and miR-155 are crucial for modulating neuroinflammation by controlling immune responses in astrocytes and microglia [34]. These miRNAs contribute to the chronic inflammatory environment that supports regenesis [35]. miRNAs like miR-134, miR-132, and miR-124 regulate synaptic plasticity and neuronal excitability by targeting genes involved in dendritic spine formation and neuronal signaling, impacting brain network stability [34, 36]. Additionally, miR-34a and miR-153 influence neuronal survival by regulating apoptotic pathways, thus controlling seizure-induced neuronal death. miRNAs such as miR-9, miR-124, miR-137, and miR-128 are involved in neurogenesis and neuronal migration, processes vital for proper cortical organization [37, 38]. Dysregulation of these miRNAs disrupts normal brain function, leading to long-term epileptogenic changes.

### miRNAs and Excitatory/Inhibitory Neurotransmission

miRNAs function by binding to complementary sequences within the 3' untranslated regions (UTRs) of target mRNAs, leading to either the degradation of the mRNA or inhibition of its translation. This post-transcriptional regulation allows miRNAs to orchestrate a wide array of cellular processes, including cell differentiation, apoptosis, and synaptic plasticity [39]. By modulating these processes, miRNAs play a crucial role in fine-tuning cellular responses to both internal and external stimuli, thus ensuring cellular homeostasis and maintaining optimal neuronal function. Given their ability to regulate gene expression in this manner, miRNAs are indispensable for the proper functioning of neuronal networks, particularly in the context of neuroplasticity and neurogenesis [40].

Among the various miRNAs implicated in brain function, miR-128 is particularly noteworthy due to its significant role in the regulation of neuronal excitability and synaptic plasticity [41]. miR-128 is abundantly expressed in the brain, where it modulates key signaling pathways, such as the ERK2 cascade, which is vital for synaptic transmission and neuronal activity. By suppressing the activation of this pathway, miR-128 ensures the maintenance of appropriate neuronal firing thresholds, preventing excessive neuronal excitability that could otherwise precipitate seizure activity [40, 41]. In the context of Parkinson’s disease (PD), for example, elevated levels of miR-128 protect dopaminergic neurons from α-synuclein-induced toxicity, thereby mitigating motor dysfunction and dyskinesia. Additionally, miR-128 plays an essential role in synaptic plasticity through its regulation of key synaptic proteins, including Synaptotagmin-1 and SNAP-25, which are involved in synaptic vesicle release and neurotransmitter signaling [42, 43]. Dysregulation of miR-128 expression has been linked to synaptic loss and cognitive decline in neurodegenerative diseases such as Alzheimer’s disease, further underscoring its pivotal role in maintaining synaptic function and neuronal integrity [34]. These studies demonstrate that miR-128 is not only crucial for neuronal function but also for the preservation of neuroplasticity and neural network stability, highlighting its potential as a therapeutic target in epilepsy and neurodegenerative diseases.

**Fig. 2:**
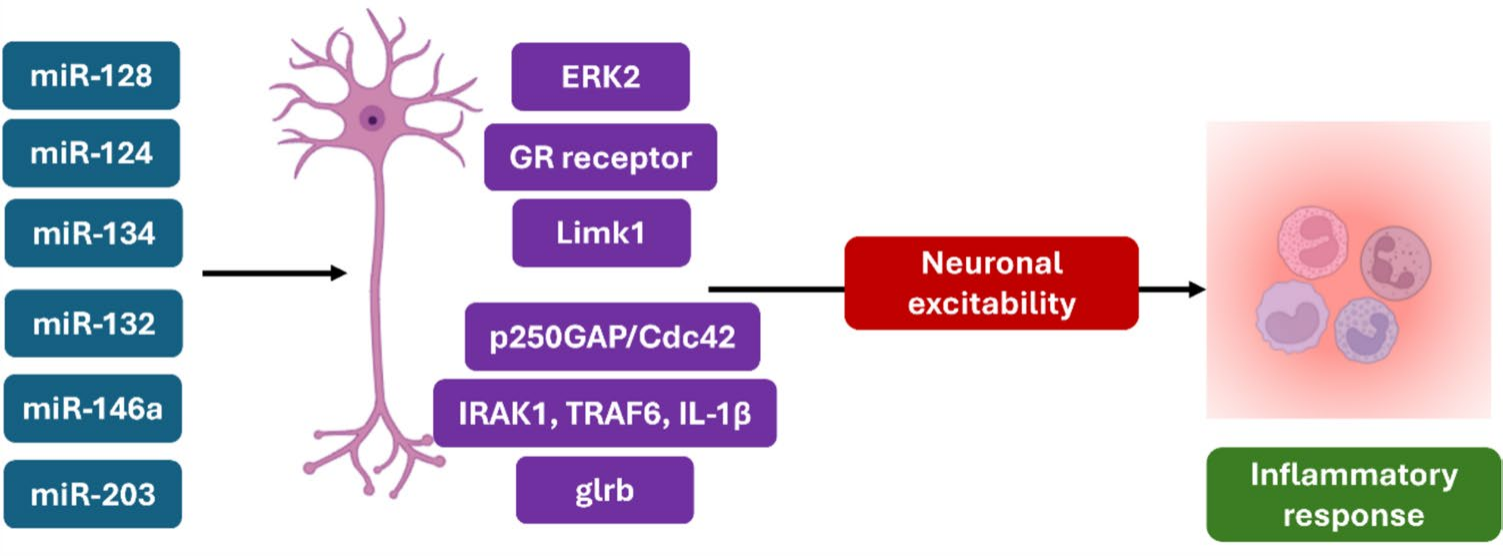
MiRNA-mediated regulation of neuronal excitability and inflammatory response. This Fig. illustrates the role of miRNAs in modulating neuronal excitability, synaptic plasticity, and inflammation in epilepsy. miR-134 regulates dendritic spine development and synaptic plasticity, while miR-132 influences neuronal excitability through the ERK2 network. Additionally, miR-146a and miR-155 are key modulators of neuroinflammation, impacting the severity and frequency of seizures. The Fig. highlights how these miRNAs contribute to epileptogenesis and their potential as therapeutic targets

A recent study revealed a strong link between miR-128 and convulsive behavior in mice. Mice with a conditional deletion of miR-128 were found to develop fatal epilepsy, which was fully prevented by treatment with antiseizure medication (ASMs). The mechanism was linked to increased excitatory transmission, and mice lacking miR-128 displayed increased spine density. Complementing these findings, overexpression of miR-128 was able to suppress seizures triggered by kainic acid, and the effects were linked to the targets of miR-128 in the extracellular signal-regulated kinase (ERK2) network [44]. In particular, miR-128 regulates the expression of numerous ion channels and transporters, as well as genes that contribute to neurotransmitter-driven neuronal excitability and motor activity [44] (Fig. 2).

Several of these genes are linked to epilepsy in humans, some of which, including the neurotransmitter GABA transporter Slc6a1, the high-affinity glutamate receptor Slc1a1, the voltage-gated sodium channels Scn2b and Scn4b, the voltage-dependent calcium channels Cacna2d3 and Cagn2, as well as the carbonic anhydrase Car7, are potential targets of clinically approved anti-seizure drugs [45]. The high abundance of ERK1/2 signaling network components among the miR-128 targets underscores the potential of this miRNA to control signaling processes associated with neuronal excitability [46]. Moreover, many of the neuronal signaling proteins and channels identified as direct miR-128 target genes are involved in regulating upstream signaling events, which can affect ERK activity. While ERK1 and ERK2 are not directly targeted by miR-128, the ERK network appears to be at the center of the miR-128-controlled signaling circuit in neurons (Fig. 2) [44].

Another miRNA for which a comprehensive link to epilepsy has been demonstrated is miR-134 [45]. miR-134 is a brain-enriched miRNA overexpressed after SE and in experimental and human epilepsy [45, 47]. Silencing miR-134 via antagomir strongly reduced intra-amygdala KA-induced seizures with long-lasting protection [45] (Fig. 2). More recent studies have confirmed these protective effects by demonstrating them in mice using the pilocarpine-induced model, providing in vivo experimental evidence to support earlier findings [48]. In vitro antiseizure effects of targeting this miRNA have also been reported [49]. The mechanism of the antiseizure effect is uncertain, but silencing miR-134 resulted in a slight decrease in hippocampal dendritic spine number in vivo, which may reduce excitability [47] (Fig. 2). This may relate to the target LIM domain kinase (Limk1), silencing of which obviated the protective effect of miR-134 in vitro [47].

miR-134 controls dendritic spine development, synaptic protein synthesis and plasticity, guidance of nerve growth cones, and has a growth-promoting effect on dendritogenesis; it induces pluripotent stem cell differentiation, exerts stage-specific modulation of cortical development, and regulates memory and cholinergic neurotransmission [46]. Membrane potential is important for maintaining the electron transport chain. miRNAs such as miR-16-5p [50], miR-195 [51], and miR-29b [52] are known to disrupt mitochondrial membrane potential and integrity, while miR-7 [53]. It is known to stabilize the membrane potential. Decreased production of energy also inhibits neurons’ abilities to transmit signals. Ultimately, disrupted transmission and the loss of neurons contribute to the cognitive impairments [54].

Importantly, accumulating evidence indicates that miRNA function in epilepsy is highly context dependent, and individual miRNAs may exert either pro-epileptogenic or neuroprotective effects depending on seizure stage, cellular localization, expression level, and brain region. A prominent example is miR-132, which displays divergent roles across experimental paradigms. In human temporal lobe epilepsy (TLE) and animal models, miR-132 expression is dynamically regulated in both neurons and glial cells, with astrocytic miR-132 overexpression attenuating pro-epileptogenic inflammatory gene expression, suggesting a neuroprotective role under specific cellular conditions [55].

In contrast, persistent miR-132 upregulation following SE has been associated with enhanced excitatory synaptic transmission, aberrant dendritic remodeling, and increased seizure susceptibility, whereas pharmacological silencing of miR-132 reduces spontaneous seizures during the chronic phase of epilepsy [56]. These findings highlight that the temporal dynamics and cellular source of miR-132 expression critically determine its functional outcome.

This functional duality is further shaped by cell-type specificity and disease stage. Neuronal miR-132 predominantly regulates synaptic proteins and plasticity-related signaling pathways, such as the p250GAP–Rac1 axis, promoting structural remodeling and excitability, whereas glial miR-132 modulates inflammatory signaling and neurotrophic factor release, influencing network homeostasis. Disease stage is also decisive: miR-132 induction during the acute seizure phase may represent a compensatory, neuroprotective response, while sustained elevation during chronic epilepsy may drive maladaptive circuit reorganization and epileptogenesis. Similar context-dependent behavior has been reported for other epilepsy-associated miRNAs, including miR-124, which exhibits both seizure-suppressive and pro-epileptogenic effects depending on timing and cellular context [57], and miR-146a, which can act as either an anti-inflammatory regulator or a contributor to chronic gliosis and seizure severity through NF-κB signaling [58]. Collectively, these observations explain apparent discrepancies across studies and underscore the necessity of interpreting miRNA dysregulation within precise temporal, cellular, and pathological frameworks to accurately define their mechanistic roles in epilepsy.

Beyond miR-128 and miR-134, several other miRNAs contribute to the regulation of neuronal excitability and network function. MiR-23a is abundantly distributed in the hippocampus and promotes myelination in the brain [59]. It has been indicated that the level of miR-23a is significantly changed in the epileptic brain of both humans and animal models [60]. Prior studies have suggested that miR-324-5p regulates multiple biological processes, such as synaptic formation and neuronal differentiation [61, 62]. The expression of miR-324-5p is upregulated during the chronic phase of the pilocarpine-induced rat model [60]. Further functional and mechanistic studies have demonstrated that, by targeting Kv4.2 of the cortex and hippocampus, a crucial regulator of neuronal excitability in the CNS [63].

Epilepsy is accompanied by aberrant excitatory neurotransmission due to dysfunctional inhibitory ion channels [64]. The inhibitory ionotropic glycine receptor (GlyR) is a transmembrane glycine-gated chloride channel. The glycine receptor-β (glrb) gene encodes GlyR subunits [65]. By targeting glrb of the hippocampus, miR-203 antagomirs reduce the frequency of spontaneous seizures during the chronic phase of the pilocarpine-induced epilepsy mouse model. In addition, in the hippocampus of epileptic mice and human epileptic brains, the expression levels of miR-203 are increased [66].

MiR-211 regulates neuronal differentiation and viability in the brain and inhibits neurite growth and branching in vitro [67]. Transgenic mice that dynamically express miR-211 in the forebrain present neuronal hyperexcitability and spontaneous seizures by targeting transforming growth factor-beta receptor type 2 (TGFβR2) [68]. TGFβR increases neuronal excitability and excitatory synaptogenesis in the setting of inflammation [69]. Moreover, when miR-211 overexpression results in spontaneous seizures, the levels of miR-134 also increase [68]. Based on the analyses in the miR-134 section, miR-134 exerts critical regulatory roles in epileptogenesis, which strengthen the roles of miR-211 in epileptogenesis [70].

Mice deficient in the miR-128–2 gene in dopamine 1 receptor-expressing neurons of the forebrain display enhanced excitability, develop a progressive seizure, and die at 2–3 months of age through effects on ion channels and transporter genes, as well as genes that contribute to neurotransmitter-driven neuronal excitability and motor activity [70].

Neuronal projections are highly enriched with miR-218 and its precursor pre-miR-218–1 [71]. During the chronic suppression of neuronal activity, the expression of miR-218 increases, and vice versa, suggesting its crucial role in regulating the homeostasis of synaptic strength. GluA2, an AMPA receptor subunit, is one of the several targets of miR-218. GluA2 is a key player in determining AMPA receptor properties, including calcium permeability, single-channel conductance, and rectification. MiR-218 positively regulates the expression of GluA2, thereby increasing glutamatergic synaptic transmission [72]. Key miRNAs involved in Neurotransmission and Epilepsy are summarized in Table 3.

**Table 3:** Functional roles of miRNAs in neurotransmission and epileptic pathophysiology

miRNA	Experimental model	Function in neurotransmission and Epilepsy	Ref
miR-128	Mouse	Regulates ion channels and transporters; overexpression suppresses seizures; targets the ERK2 network; deletion causes fatal epilepsy	[44]
miR-134	Human	Controls dendritic spine development and synaptic plasticity; silencing reduces seizures; targets Limk1	[73, 74]
miR-16-5p	In vitro	Disrupts mitochondrial membrane potential, leading to impaired signal transmission	[50]
miR-195	Mouse	Disrupts mitochondrial membrane potential and integrity, contributing to cognitive impairment	[51]
miR-29b	Rat	Alters mitochondrial function; associated with reduced energy production and impaired neurotransmission	[52]
miR-7	In vitro	Stabilizes mitochondrial membrane potential and supports neuronal energy maintenance	[53]
miR-23a	Rat	Promotes myelination; altered levels in the epileptic hippocampus	[59, 60]
miR-324-5p	Rat	Targets Kv4.2, regulating neuronal excitability; upregulated in chronic epilepsy model	[61]
miR-203	Mouse	Targets glrb; inhibition reduces seizures in epilepsy models	[66]
miR-211	Mouse	Regulates neuronal differentiation; overexpression causes spontaneous seizures via the TGFβR2 pathway	[67, 68]
miR-218	Mouse	Regulates GluA2 and synaptic strength; upregulation enhances glutamatergic transmission	[71]

ERK2: Extracellular-Regulated Kinase 2, Limk1: LIM domain kinase 1, glrb: Glycine Receptor Beta, TGFβR2: Transforming Growth Factor, Beta Receptor II, GluA2: glutamate AMPA receptor

### miRNAs and Neuroplasticity

Synaptic plasticity is essential for memory and learning. Mitochondria provide the energy necessary for plasticity and aid in calcium buffering that occurs at the synapse. Mitochondrial miRNAs have been found to be involved in synaptic plasticity. miR-132 [75] has a role in regulating short-term plasticity, while miR-484 impacts long-term plasticity (Table 4) [76].

**Table 4:** Functional overview of miRNAs implicated in neuroplasticity and Epilepsy

miRNA	Experimental model	Function in Neuroplasticity	Ref
miR-132	Mouse/Rat	Regulates dendritic growth and spine formation; involved in short-term synaptic plasticity; targets p250GAP; increased in epilepsy models	[70, 82]
miR-134	Mouse/Rat	Regulates spine morphology and dendritic growth; affects Limk1, CREB, and Pumilio-2; involved in synaptic downscaling and plasticity; upregulated in epilepsy	[47, 73, 74, 80]
miR-484	Human/In vitro	Influences long-term synaptic plasticity via mitochondrial regulation	[49, 76]
miR-219	Rat	Regulates synaptic plasticity, oligodendrocyte differentiation, and myelination; downregulated in epilepsy	[85–87]
miR-139-5p	Rat/Human	Negatively regulates NMDAR subunits NR2A and NR2B; affects synaptic plasticity and neuronal excitability	[89]

p250GAP: Rho GTPase activating protein 32, CREB: cAMP Response Element-Binding Protein, NMDAR: N-methyl-D-aspartate receptor

Another activity-dependent miRNA is miR-134; it was discovered as a dendritic miRNA of hippocampal neurons in 2006 [47], and a recent paper demonstrates the loop sequence of its pre-miRNA form is necessary for the interaction with DHX36 and the accumulation in dendrites [77]. At the synapse, miR-134 regulates spine morphology by local inhibition of Limk1 mRNA, a cytoskeleton regulator. Stimulation with brain-derived neurotrophic factor (BDNF), usually secreted by neuronal activity, releases Limk1 from miR-134 inhibition, promoting Limk1 protein translation. Indeed, overexpression in vitro of miR-134 reduces dendritic spine volume and synaptic strength of hippocampal neurons, while miR-134 silencing has opposite effects [47].

In addition, miR-134 also targets Limk1 in primary hippocampal neurons. Limk1 is also critical for synaptic transmission, plasticity, and memory formation. Antagomirs targeting miR-134 increased Limk1 levels during the acute phase of the pilocarpine-induced epilepsy rat model [78]; however, although these results suggest that miR-134 targets tulp1 and Limk1 in epileptogenesis, they do not reveal the direct effects of miR-134 targeting on epileptogenesis [70].

Additional in vivo analysis demonstrated that miR-134 targets also the transcription factor CREB [79]. In turn, miR-134 was found to be negatively regulated by sirtuin 1 (SIRT1) in cooperation with the transcription factor YY1 during learning and memory. SIRT1 KO mice, which lack this mechanism, have LTP impairments in CA1 neurons and deficits in memory performance due to the reduction of CREB and BDNF. Recently, the role of miR-134 has also been studied in the context of disease mechanisms since it has been demonstrated that miR-134 is upregulated in experimental and human epilepsy [73, 79]. Interestingly, miR-134 downregulation by antagomir intrahippocampal injection protected against seizure induction and decreased spine density of hippocampal CA3 neurons caused by kainate, probably acting via derepression of Lmk1. Because of its synaptic actions, miR-134 has also been studied in homeostatic plasticity. miR-134 was found to be necessary for both synapse elimination and structural rearrangements leading to synaptic downscaling [80].

MiR-134 downregulation of Pumilio-2, a RBP involved in miRNA transport and translation inhibition, is required for miR-134-mediated homeostatic synaptic depression in response to chronic activity. Using a compartmentalized culture system, the authors demonstrated that this inhibition occurs specifically in the dendritic compartment. Given that AMPA internalization is a necessary step of homeostatic plasticity, they investigated the possible contribution of miR-134 in this process, identifying polo-like kinase 2 as a novel target of Pumilio-2 involved in the control of GluA2 surface expression. This is a novel pathway of homeostatic plasticity that stabilizes neuronal circuits in response to increased network activity, and future studies could be interesting to investigate how this pathway contributes to miR-134's neuroprotective role in epilepsy, searching for a possible therapeutic use of antagomirs [81].

MiR-132 regulates the dendritic growth and spine formation of newborn neurons in the adult hippocampus and cognitive capacity in the brain [70, 82]. Increased miR-132 levels are found in the miRNA expression profile of kainic acid–induced epileptic mice, and depletion of miR-132 reduces seizure-induced neuronal death [56]. A prior mechanistic study suggested that miR-132 also plays an important role in synaptic plasticity by targeting p250GAP [83]. p250GAP is an important cytoskeletal regulator that interacts with various synaptic proteins and regulates multiple neurobiological processes [84], such as axonal growth and dendritic spine morphology [70].

MiR-219, another brain-specific miRNA expressed in both rodents and human brains, is known to play a critical role in the regulation of neural activity, especially oligodendrocyte differentiation, myelination, and synaptic plasticity [85, 86]. The level of miR-219 is found to be downregulated in the brains of SE rat models and cerebrospinal fluid samples of patients with epilepsy, indicating its role in the pathogenesis of epilepsy [87]. According to a previous study, miR-219 can directly target CaMKIIγ to negatively influence the function of NMDA receptors [88]. In contrast to their expression in controls, another study found an inverse relationship between miR-219 and NMDA-NR1 expression in the hippocampus and amygdala [1].

The role of miR-139-5p in epilepsy emerges as a crucial regulatory element. It negatively regulates the NMDAR subunits, particularly NR2A and NR2B. By restoring the levels of miR-139-5p using its mimetic, a corresponding change was observed in the NR2A expression. This interplay suggests that miR-139-5p exerts a regulatory effect on NMDAR activity, contributing to neuronal hyper-excitation and changes in synaptic plasticity implicated in epilepsy [89]. According to the results of the previous study, the expression of miR-139-5p was associated with cortical development, and upregulation of miR-139-5p may attenuate the damaged cortex. This could be because miR-139-5p regulates neuronal migration in the cortex by targeting Lis1 [90]. Furthermore, by inhibiting the human growth transformation-dependent protein, miR-139-5p agomir reduces brain damage [90].

### miRNAs and Hypothalamic–Pituitary–Adrenal Axis

Epilepsy is a complex neurological disorder characterized by the spontaneous recurrence of seizures due to abnormal neuronal activity. Over the past decade, increasing attention has been directed toward the molecular underpinnings of epilepsy, with miRNAs emerging as critical players in the regulation of neuronal excitability and inflammation. In the context of epilepsy, specific miRNAs have been found to be dysregulated in both human patients and experimental models, suggesting their active participation in epileptogenesis, the process by which a normal brain develops epilepsy. These dysregulated miRNAs may either promote or suppress seizure development by affecting genes involved in neuronal signaling, apoptosis, and glial activation [91]

Among the miRNAs implicated in epilepsy, miR-132, miR-146a, and miR-134 are notably prominent [92]. For instance, miR-132, which is associated with synaptic plasticity and neurite outgrowth, is typically upregulated following seizures and may exacerbate excitatory signaling in neurons. Similarly, miR-146a is involved in regulating inflammatory pathways, especially within astrocytes and microglia, and its persistent upregulation has been associated with chronic neuroinflammation in epileptic tissue [93]. This inflammatory response is a known contributor to seizure susceptibility and frequency. The overexpression of miR-134, on the other hand, has been shown to negatively impact dendritic spine morphology, which can lead to altered synaptic strength and connectivity, two core features in the development of hyperexcitable neural circuits [94].

In parallel to these neural mechanisms, the role of the hypothalamic–pituitary–adrenal [95] axis in epilepsy has gained increasing attention. The HPA axis is the body’s central stress response system, and stress is a well-established precipitating factor for seizures in many individuals with epilepsy [96]. Dysregulation of the HPA axis, such as excessive release of corticotropin-releasing hormone (CRH) or glucocorticoids, can lower the seizure threshold and amplify neuronal excitability. miRNAs have been identified as key regulators of this axis (Table 5). For example, miR-124, which is abundantly expressed in the brain, negatively regulates the expression of the glucocorticoid receptor (GR) [97]. Reduced GR expression impairs the negative feedback regulation of the HPA axis, resulting in prolonged exposure to cortisol during stress, which in turn can exacerbate seizure activity and contribute to the maintenance of an epileptic state [98].

**Table 5:** MiRNAs linking epilepsy and HPA axis dysfunction: Biological functions and mechanisms

miRNA	Biological function	Experimental model	Role in Epilepsy or HPA axis	Ref
miR-132	Regulates synaptic plasticity and neurite outgrowth	Mouse	Upregulated after seizures; enhances excitatory signaling, contributing to neuronal hyperexcitability	[92]
miR-146a	Modulates inflammatory responses, especially in astrocytes and microglia	Human/Mouse	Promotes chronic neuroinflammation, a key factor in seizure frequency and susceptibility	[93, 101]
miR-134	Influences dendritic spine development and synaptic strength	Mouse	Overexpression disrupts neural connectivity; inhibition reduces seizure severity and neuronal damage	[94, 100]
miR-124	Negatively regulates GR expression	Rat	Reduced GR expression impairs HPA axis feedback, leading to prolonged cortisol exposure and seizure aggravation	[97, 98, 101]
miR-375	Targets pituitary hormone-related genes	Rat	Alters hormone secretion, potentially disrupting HPA axis function and stress responses	[99]
miR-34c	Regulates CRH in the hypothalamus	Mouse	Affects CRH production and HPA axis activation, increasing stress-induced vulnerability to seizures	[99]

HPA: Hypothalamic–pituitary–adrenal axis, GR: glucocorticoid receptor, CRH: corticotropin-releasing hormone

Further supporting this connection, miR-375 and miR-34c have been shown to modulate the HPA axis at multiple levels. miR-375, for instance, targets pituitary-specific genes involved in hormone secretion, while miR-34c modulates CRH expression in the hypothalamus [99]. Altered expression of these miRNAs in stress-related disorders hints at a broader role in neuropsychiatric and neurological conditions, including epilepsy. A disruption in their normal expression may result in dysregulated hormone levels, impairing the body's ability to cope with stress and making the brain more vulnerable to seizure generation and propagation. Moreover, chronic activation of the HPA axis due to sustained stress can lead to structural changes in hippocampal neurons, a region critically involved in the pathogenesis of temporal lobe epilepsy [98].

What makes miRNAs especially relevant in this context is their ability to fine-tune multiple genes simultaneously, enabling them to exert widespread effects on both neuronal and endocrine functions. This multifaceted regulation positions them at a unique intersection between neurological activity and systemic stress responses. Targeting miRNAs could, therefore, offer a dual therapeutic benefit: modulating aberrant neural activity while also normalizing HPA axis dysfunction. Preclinical studies using miRNA mimics or inhibitors (antagomirs) have shown promising results in reducing seizure frequency and severity by restoring homeostatic gene expression patterns. For example, silencing miR-134 in animal models has been shown to reduce seizure-induced neuronal damage and improve behavioral outcomes, hinting at the therapeutic viability of such approaches [100].

From a translational perspective, miRNAs could also serve as biomarkers for epilepsy diagnosis, prognosis, or treatment response. Since they are stable in body fluids like blood and cerebrospinal fluid, miRNA profiles could reflect both CNS changes and peripheral stress responses. For instance, elevated levels of circulating miR-146a or altered miR-124 expression could indicate a state of neuroinflammation or HPA axis dysregulation, respectively. This offers clinicians a non-invasive method to monitor disease progression or evaluate treatment efficacy. However, more clinical trials and longitudinal studies are needed to validate these findings and determine how best to integrate miRNA-targeted therapies into routine clinical practice [101].

### miRNAs and Inflammatory Response

One of the most extensively studied miRNAs in this context is miR-146a, which plays a pivotal role in the negative feedback regulation of innate immune signaling pathways. It is typically induced following the activation of the nuclear factor kappa-light-chain-enhancer of activated B cells (NF-κB) pathway, a major transcriptional regulator of inflammation. In epilepsy, persistent upregulation of miR-146a has been reported in human epileptic tissue and animal models, suggesting a maladaptive response that fails to resolve chronic neuroinflammation. While miR-146a initially acts to dampen the pro-inflammatory signals by targeting IRAK1 and TRAF6, key signaling intermediates, its continued overexpression may impair immune resolution and contribute to sustained glial activation and seizure recurrence (Table 6) [102–104].

**Table 6:** Inflammation-associated miRNAs in Epilepsy: Functional insights and target pathways

miRNA	Function in inflammatory response & Epilepsy	Experimental model	Key targets/effects	Ref	
miR-146a	Regulates innate immune signaling; persistently upregulated in epilepsy, contributing to chronic inflammation	Human/Rat	Targets IRAK1 and TRAF6; modulates NF-κB pathway		[102–104]
miR-155	Pro-inflammatory; promotes microglial activation and cytokine production; enhances seizure severity	Mouse	Increases TNF-α and IL-1β levels	[105]	
miR-124	Anti-inflammatory; usually downregulated in epilepsy; restoring its levels improves neuronal survival	Rat	Suppresses microglial activation; limits neuroinflammation	[106, 107]	

IRAK1: Interleukin-1 receptor-associated kinase 1, TRAF6: Tumor Necrosis Factor receptor-associated factor 6, NF-κB: Nuclear factor kappa-light-chain-enhancer of activated B cells, TNF-α: Tumor Necrosis Factor-alpha, IL-1β: Interleukin-1 beta

Another miRNA implicated in epilepsy-related inflammation is miR-155, a pro-inflammatory miRNA known for its role in activating microglia and amplifying cytokine production. Elevated miR-155 levels have been observed in experimental models of SE and are associated with increased levels of TNF-α and IL-1β, two cytokines strongly linked to epileptic activity. Inhibition of miR-155 in such models has been shown to reduce neuroinflammation and decrease seizure severity, underscoring its pathogenic potential [105]. These findings highlight miR-155 as both a mediator and a potential biomarker of seizure-induced inflammatory states.

In epilepsy, reduced levels of miR-124 have been detected, potentially leading to unchecked microglial activity and sustained inflammation. Restoring miR-124 expression in animal models of epilepsy has been associated with reduced glial activation and improved neuronal survival, suggesting a therapeutic angle that warrants further exploration [106, 107].

Overall, the interplay between miRNAs and inflammation in epilepsy is complex and context-dependent. Some miRNAs exacerbate the inflammatory milieu that fosters epileptogenesis, while others attempt to restore homeostasis. Understanding this balance is crucial not only for deciphering the molecular basis of epilepsy but also for developing miRNA-based therapeutic strategies. Such therapies could offer precision targeting of neuroinflammatory processes, potentially preventing or alleviating seizures without the broad side effects of current anti-inflammatory or anticonvulsant drugs [57, 108].

### Comparative Insights Between Animal Models and Human Clinical Data

Comparative analyses between animal models and human studies reveal both convergence and divergence in miRNA expression associated with epilepsy. Several miRNAs exhibit conserved regulatory patterns across species, reflecting shared molecular mechanisms in the development of epilepsy. For example, elevated miR-134 expression was initially identified as a driver of dendritic remodeling and seizure susceptibility in mice [73, 109]. Similarly increase was observed in the plasma of patients with temporal lobe epilepsy [110]. Likewise, miR-146a, a modulator of the TRAF6/IRAK1–NF-κB pathway, shows parallel upregulation in rodent hippocampi and human epileptic tissue, indicating conserved neuroinflammatory signaling [111]. However, notable interspecies discrepancies persist. miR-134 robustly suppresses LIMK1 in rodents but interacts weakly with the human 3′UTR [112, 113], and miR-128 targets ERK2 in mice but regulates SCN1A and KCNA1 in humans [114]. Similarly, miR-181a shows stage-dependent fluctuations in rodents yet stable expression in patients, underscoring the necessity for humanized and patient-derived models to enhance translational accuracy in miRNA-based epilepsy research [72].

### miRNAs and Therapeutic Intervention in Epilepsy

**Fig. 3:**
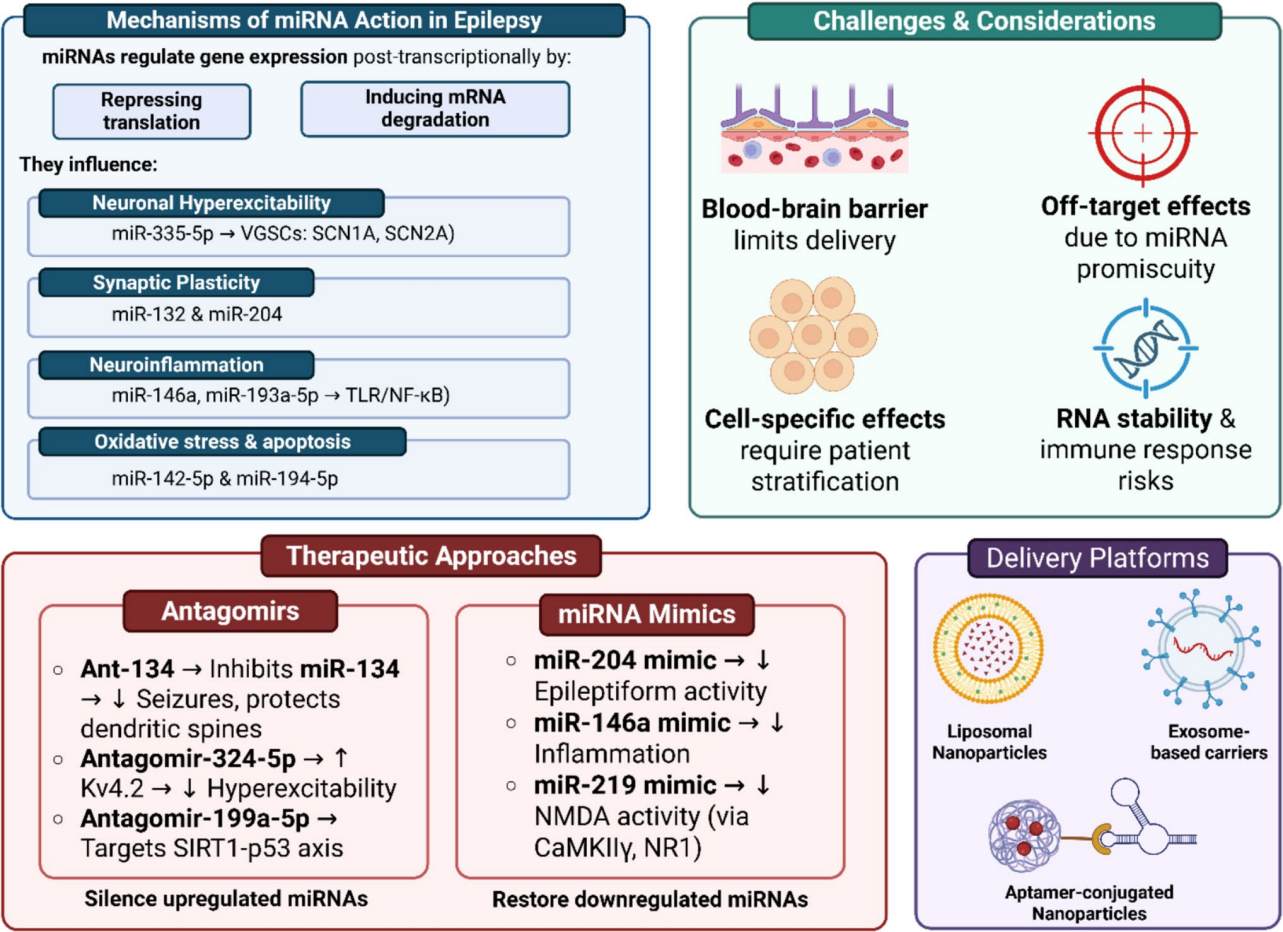
miRNAs and therapeutic intervention in epilepsy. This Fig. illustrates the therapeutic potential of miRNAs in epilepsy. It highlights their regulatory roles in epileptogenic through modulation of neuronal excitability, synaptic plasticity, inflammation, oxidative stress, and apoptosis. Key examples include miR-134, miR-335-5p, and miR-146a, among others. Therapeutic strategies involve antagomirs (to silence overexpressed miRNAs) and mimics (to restore downregulated ones), with preclinical evidence supporting seizure reduction and neuroprotection. Delivery platforms such as nanoparticles and intranasal routes are emphasized, alongside challenges like blood–brain barrier penetration, off-target effects, and translational limitations

MiRNAs are increasingly recognized as critical molecular regulators in the pathogenesis of epilepsy, offering highly specific, multifaceted avenues for therapeutic intervention beyond the limitations of current ASMs (Fig. 3). Epilepsy affects over 70 million individuals globally, with nearly 30% of patients classified as drug-resistant epilepsy [105], a condition where seizures persist despite optimal pharmacological treatment. This urgent clinical need has catalyzed the exploration of novel molecular targets, with miRNAs emerging as central players due to their role in fine-tuning gene expression post-transcriptionally [92].

Functionally, miRNAs exert regulatory control by binding to complementary sequences on target mRNAs, leading to either mRNA degradation or translational repression. In epilepsy, specific miRNAs exhibit consistent patterns of dysregulation, influencing diverse pathophysiological mechanisms such as neuronal hyperexcitability, synaptic reorganization, inflammation, oxidative stress, and programmed cell death. For instance, miR-134, enriched in the dendritic compartments of hippocampal neurons, regulates dendritic spine morphology by targeting Limk1 and is markedly upregulated in temporal lobe epilepsy [62]. Its inhibition via Ant-134 has shown robust anticonvulsant and neuroprotective effects across multiple preclinical models. Similarly, miR-335-5p modulates voltage-gated sodium channels (VGSCs), including *SCN1A*, *SCN2A*, and *SCN3A*, and its downregulation correlates with increased neuronal excitability in PTZ-induced seizure models. Other miRNAs, such as miR-132, miR-204, and miR-219, have been linked to synaptic plasticity, ERK1/2-CREB signaling, and NMDA receptor regulation, respectively, while miR-146a and miR-193a-5p are intimately involved in controlling pro-inflammatory pathways via the TLR/NF-κB axis [73, 92, 115]. These molecular mechanisms, expression patterns, and intervention strategies are systematically summarized in Table 7.

**Table 7:** Therapeutic targeting of dysregulated miRNAs in epilepsy—mechanisms, models, and preclinical evidence

miRNA	Expression in Epilepsy	Target genes/Pathways	Therapeutic strategy	Experimental model	Model/System	Key findings	Ref
miR-134	Upregulated	Limk1 (dendritic spine morphology)	Antagomir (Ant-134)	Mouse/Rat	TLE rodent model	Reduced seizure frequency; improved dendritic structure	[73]
miR-335-5p	Downregulated	*SCN1A*, *SCN2A*, *SCN3A* (VGSCs)	miRNA mimic	Rat	PTZ rodent model	Lowered neuronal excitability and sodium current density	[126]
miR-324-5p	Upregulated	Kv4.2 potassium channels	Antagomir	Rat	SE and chronic epilepsy models	Reduced seizure frequency and interictal spikes; hormone-influenced expression	[119]
miR-132	Upregulated	p250GAP/Cdc42 (spine and synaptic plasticity)	Antagomir	Rat	LI-PC rat model	Reduced dendritic branching; suppressed spontaneous seizures	[120]
miR-204	Downregulated	ERK1/2-CREB signaling	miRNA mimic	Rat	Mg^2^⁺-free SE model	Reduced epileptiform discharges	[127]
miR-219	Downregulated	CaMKIIγ and NR1 (NMDA receptor regulation)	miRNA mimic	Rat	KA-induced epilepsy	Restored EEG normalcy; reduced NMDA-related excitotoxicity	[128]
miR-146a	Downregulated	IRAK1, TRAF6, IL-1β (inflammatory signaling)	miRNA mimic	Rat/Mouse	TLE and immature rat SE models	Suppressed neuroinflammatory markers and seizure severity	[129]
miR-193a-5p	Upregulated	IL-1β, IL-6, Bax/Bcl-2 (inflammation and apoptosis)	Antagomir	Rat	LI-PC rodent model	Decreased inflammation and hippocampal neuron apoptosis	[130]
miR-199a-5p	Upregulated	*SIRT1*, acetylated *p53*, cleaved caspase-3	Antagomir	Rat	SE model	Reduced seizure-like EEG spikes and neuron loss	[117]
miR-142-5p	Upregulated	Miro1, Trak1/2 (mitochondrial transport and energy balance)	Antagomir	Mouse	SE and TLE models	Improved mitochondrial health; reduced oxidative stress and apoptosis	[121]
miR-210	Downregulated	α7nAChR pathway and p-Akt signaling	miRNA mimic	Rat	Ischemia/reperfusion injury model	Reduced apoptosis and oxidative damage; promoted neuroprotection	[131]
miR-145	Upregulated	Caspase-9 (apoptosis regulation)	Antagomir	Rat	LI-PC rat model	Improved hippocampal neuron survival and memory-related functions	[132]
miR-137	Downregulated	Pyramidal neuron excitability; seizure latency	miRNA mimic	Mouse/Rat	TLE and PTZ rodent models	Increased latency to seizure onset; reduced kindling severity	[133]
miR-194-5p	Upregulated	PTGS2, GPX4 (ferroptosis and oxidative stress)	Antagomir	Rat	TLE rodent model	Lowered iron toxicity and lipid peroxidation; reduced seizure scores	[122]

Limk1: LIM domain kinase 1, SCN1A: Sodium voltage-gated channel alpha subunit 1, VGSCs: Voltage-Gated Sodium Channels, IL-1β: Interleukin-1 beta, IL-6: Interleukin 6, Bax: Bcl-2-associated X protein, Bcl-2: B-cell lymphoma 2, SIRT1: Sirtuin 1, Miro1: Mitochondrial Rho GTPase 1, Trak1/2: Trafficking Kinesin Protein 1/2, α7nAChR: Αlpha 7 nicotinic acetylcholine receptors, PTGS2: Prostaglandin-endoperoxide Synthase 2, GPX4: Glutathione Peroxidase 4

Therapeutic strategies to harness the potential of miRNAs in epilepsy involve two principal modalities: miRNA mimics and antagomirs. Mimics are synthetic RNA duplexes designed to restore the function of downregulated miRNAs, whereas antagomirs are chemically modified antisense oligonucleotides used to silence overexpressed miRNAs. A leading example is Ant-134, which silences miR-134 and has demonstrated efficacy in suppressing spontaneous seizures, reducing hippocampal damage, and restoring dendritic architecture. Similar successes have been observed with antagomirs targeting miR-199a-5p, miR-324-5p, and miR-135a—each acting through distinct pathways such as SIRT1-p53 regulation, Kv4.2 channel expression, and synaptic plasticity modulation. Conversely, miRNA mimics such as those for miR-204, miR-146a, and miR-137 have been shown to decrease epileptiform discharges, attenuate hippocampal inflammation, and enhance inhibitory neurotransmission, respectively. Notably, intranasal and intracerebroventricular (ICV) delivery routes have been employed successfully in vivo, and emerging delivery platforms, including nanoparticle and exosome-based carriers, aim to overcome the challenges of BBB permeability and off-target effects [58, 116, 117].

The preclinical evidence for miRNA-based interventions is substantial and expanding. For example, miR-324-5p antagomir therapy upregulates Kv4.2 potassium channels, thereby curbing neuronal hyperexcitability and reducing interictal spikes and spontaneous seizures in both acute and chronic epilepsy models. In female mice, hormone-modulated expression of miR-324-5p indicates that sex-specific responses to miRNA-targeted therapies may exist. miR-132 inhibition, acting via the Rho GTPase family and p250GAP-Cdc42 pathway, has been shown to reduce seizure frequency and normalize dendritic spine density. Meanwhile, miR-219 mimics target CaMKIIγ and NR1 to reduce NMDA receptor activity and restore EEG normalcy [118–120].

One of the most compelling features of miRNA-based therapy is the ability to modulate multiple downstream targets and biological pathways through a single molecule. For example, miR-142-5p inhibition not only reduces seizure severity but also enhances mitochondrial function, improves oxidative stress profiles (e.g., ↓ ROS, ↓ MPO, ↑ SOD), and attenuates apoptosis (↓ Bax, ↑ Bcl-2). miR-194-5p, recently implicated in ferroptosis and neuronal lipid peroxidation, when inhibited, significantly reduces seizure scores, iron content, and oxidative stress biomarkers in TLE models. These findings suggest that certain miRNAs could function as master regulators of epileptogenesis, offering system-level modulation that pharmacological monotherapies fail to achieve [121, 122].

However, translational challenges remain before miRNA-based therapeutics can be fully integrated into clinical practice. Foremost among these are issues related to the delivery of RNA-based agents across the BBB, the risk of off-target effects due to miRNAs' promiscuity, and ensuring the stability and bioavailability of synthetic RNA molecules in vivo. Additionally, miRNAs often act in a cell-type and context-specific manner, necessitating careful validation in human tissues and stratified patient cohorts. Regulatory hurdles surrounding the long-term safety and immunogenicity of RNA therapeutics also demand rigorous investigation.

For instance, a recent study investigating lipid nanoparticle (LNP)-mediated delivery of miR-124 for neuroinflammation found that while the formulation showed therapeutic efficacy in mice, achieving sufficient CNS penetration after systemic administration required extensive optimization of ionizable lipids, highlighting that even advanced LNP platforms face significant hurdles in crossing the BBB for miRNA-based therapies [123]. Furthermore, successful cases like antagomir-134-mediated seizure suppression in mice often depend on pathological states that transiently compromise the BBB, such as during status epilepticus. This reliance on a temporary and clinically limited window exposes the critical gap in the field: achieving robust and consistent delivery of miRNA therapeutics to the brain under normal physiological conditions for chronic treatment applications remains an unresolved and fundamental hurdle [124].

To address these limitations, current research focuses on advanced delivery systems (e.g., liposomal carriers, aptamer-conjugated nanoparticles), combination regimens with existing ASMs for synergistic efficacy, and biomarker-guided patient selection. The recent identification of circulating miRNAs in serum and Cerebrospinal Fluid (CSF) (e.g., miR-301a-3p, miR-194-5p) also opens avenues for companion diagnostics, allowing miRNAs modulation to be tailored to individual molecular profiles [92, 125].

## Limitations of miRNA-Based Therapeutics: Dosing, Safety, and Off-Target Effects

Although miRNA-based therapies have shown encouraging neuroprotective effects in preclinical epilepsy models, their clinical translation remains limited by challenges in dosing, safety, and species specificity. Optimizing dosage is particularly challenging, as miRNAs regulate extensive gene networks, and even minor changes in expression can disrupt neuronal balance. For instance, miR-134 antagomirs reduced hippocampal damage and seizure frequency in mice, but excessive inhibition impaired dendritic remodeling, highlighting a narrow therapeutic window [113, 134]. Similarly, overexpression of miR-132 or miR-124 paradoxically triggered neuroinflammation and excitotoxicity by suppressing protective pathways [135]. These findings underscore the dual, context-dependent nature of miRNAs, where modulation can either alleviate or exacerbate pathology depending on dose, disease phase, or cellular environment. Systemic delivery adds further complications due to rapid degradation, poor blood–brain barrier penetration, and peripheral accumulation leading to immune activation and hepatotoxicity [136].

Species-specific differences in miRNA–mRNA interactions also hinder the translation of findings from animal models to humans. For example, miR-128 targets the ERK2 signaling pathway in mice but primarily regulates ion channel genes such as SCN1A in human neurons; miR-134 controls dendritic architecture through LIMK1 suppression in rodents but binds weaker, divergent UTR sites in humans; and miR-146a exhibits altered kinetics and reduced immunomodulatory efficiency in human astrocytes compared to rodents [137, 138]. Likewise, miR-9 regulates *REST/NRSF* signaling in mice but influences Notch1 and TLX in humans, leading to distinct neurodevelopmental outcomes. Such interspecies divergence contributes to inconsistent efficacy and unpredictable target engagement in clinical translation [134].

Additionally, off-target gene regulation and insufficient long-term safety data remain significant obstacles. miR-146a mimics suppressed NF-κB-mediated inflammation but also silenced genes essential for synaptic repair, while miR-199a, miR-128, and miR-335 altered apoptosis and oxidative-stress pathways beyond epileptogenic circuits [72, 139].

## Potential Side Effects of Chronic miRNA Therapy

While miRNA-based therapeutics have shown robust anticonvulsant and neuroprotective effects in acute and sub-chronic models of epilepsy, their long-term safety under chronic dosing conditions remains poorly defined. Sustained manipulation of miRNA expression can remodel extensive gene networks involved in neuronal excitability, gliotransmission, and synaptic stability, resulting in transcriptomic reprogramming that extends beyond intended targets [140]. Such prolonged modulation risks disturbing the delicate equilibrium between excitation and inhibition within epileptogenic circuits and may promote maladaptive plasticity or gliotic remodeling [141]. For instance, chronic inhibition of miR-134, though protective acutely, can alter dendritic spine density and downstream BDNF-CREB signaling during extended suppression [142]. Likewise, repetitive dosing of chemically stabilized oligonucleotides, particularly phosphorothioate or locked nucleic acid (LNA) analogues, has been shown to trigger innate immune activation through Toll-like receptor pathways and type I interferon signaling, leading to neuroinflammation, microgliosis, and compromised blood–brain barrier integrity [143, 144]. These immune-mediated responses could exacerbate seizure susceptibility and diminish the therapeutic window of miRNA-based interventions in chronic epilepsy.

Additionally, repeated systemic or intracerebral delivery introduces risks of cumulative toxicity and altered pharmacokinetics. Lipid- or polymer-based carriers, when administered chronically, may accumulate within hepatic, renal, or glial compartments, altering clearance and eliciting low-grade inflammatory or oxidative stress responses [144]. Longitudinal non-clinical studies have underscored the need to define time–dose–effect relationships and assess the reversibility of miRNAs modulation through chronic exposure paradigms [145]. To ensure translational safety, future chronic dosing models in epilepsy should incorporate integrated pharmacogenomic, neurobehavioral, and immunotoxic assessments alongside controlled-release or biodegradable delivery systems that restrict exposure to epileptogenic foci. Establishing these chronic-safety parameters will be essential to translate miRNA therapeutics from acute experimental efficacy to sustained clinical benefit in refractory epilepsy [145, 146].

## Clinical Importance of miRNAs (Diagnosis & Prognosis)

**Fig. 4:**
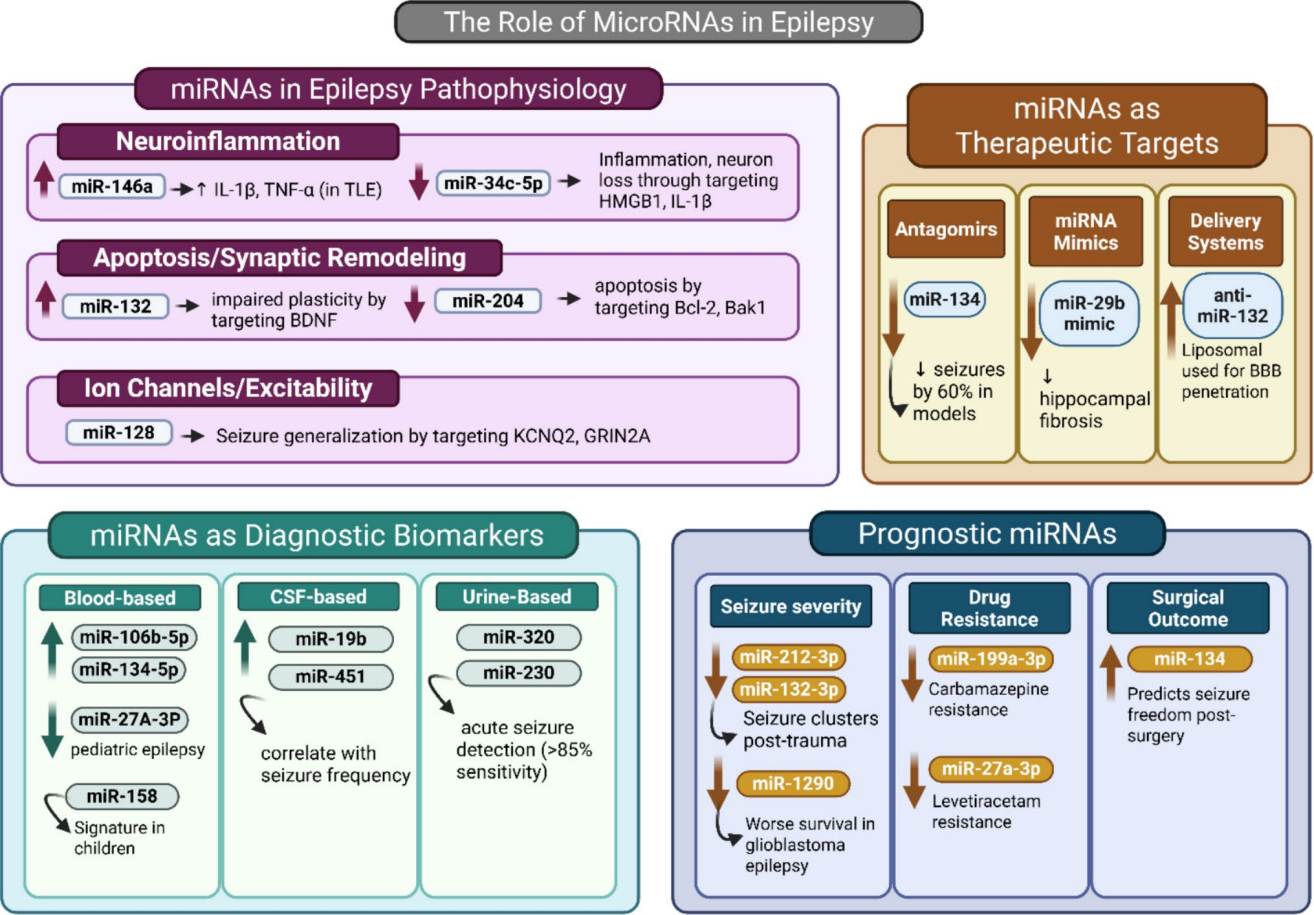
The Role of miRNAs in Epilepsy: Diagnostic and Prognostic Biomarkers. This Fig. summarizes the multifaceted roles of dysregulated miRNAs in epilepsy, categorized by their involvement in neuroinflammation (e.g., miR-146a, miR-34c-5p), neuronal apoptosis and synaptic remodeling (e.g., miR-132, miR-204), and ion channel regulation (e.g., miR-128). Diagnostic applications highlight circulating miRNAs detectable in blood, cerebrospinal fluid, and urine for epilepsy classification and severity assessment. Prognostic markers predict seizure recurrence, drug resistance, and surgical outcomes (e.g., miR-212-3p, miR-27a-3p, miR-134). The Fig. also outlines technical considerations for miRNA detection and therapeutic strategies using antagomirs, mimics, and nanoparticle delivery systems for seizure control

Epilepsy affects over 70 million people worldwide, with approximately 4.9 million new cases annually [92, 147]. Despite advances in treatment, 30% of patients develop drug-resistant epilepsy, highlighting the need for innovative diagnostic and prognostic tools [147, 148]. miRNAs have emerged as promising biomarkers due to their stability in biofluids, disease-specific dysregulation, and involvement in epileptogenesis [92, 148]. Understanding the pathophysiological basis of miRNA dysregulation is crucial for interpreting their diagnostic and prognostic roles (Fig. 4).

Several miRNAs are involved in neuroinflammation; miR-146a is upregulated in TLE, a type of epilepsy where seizures originate in the temporal lobes of the brain. It modulates the Toll-like receptor 4 (TLR4) pathway and NF-κB signaling (Table 8). This promotes the release of pro-inflammatory cytokines (e.g., IL-1β, TNF-α), exacerbating neuronal hyperexcitability [149]. miR-34c-5p is downregulated in drug-resistant epilepsy increases high-mobility group protein B1 (HMGB1) and IL-1β, amplifying neuroinflammation and neuronal loss [150].

**Table 8:** Key miRNAs in epilepsy pathophysiology

miRNA	Experimental model	Pathogenic process	Target genes/proteins	Functional outcome	Ref
miR-146a	Human/Rat	Neuroinflammation	TLR4, NF-κB	↑ IL-1β, TNF-α	[149]
miR-204	Rat	Apoptosis	Bcl-2, Bak1	↑ Neuronal survival	[152]
miR-132	Mouse/Rat	Synaptic plasticity	BDNF	Impaired neurogenesis	[149, 151]
miR-128	Mouse	Ion channel function	KCNQ2, GRIN2A	↑ Neuronal excitability	[92, 154]

TLR4: Toll-like receptor 4, NF-κB: Nuclear factor kappa-light-chain-enhancer of activated B cells, IL-1β: Interleukin-1 beta, TNF-α: Tumor Necrosis Factor-alpha, Bcl-2: B-cell lymphoma 2, Bak1: BCL2 Antagonist/Killer 1, BDNF: Brain-Derived Neurotrophic Factor, KCNQ2: Potassium voltage-gated channel subfamily member 2

Other miRNAs are involved in neuronal apoptosis and synaptic remodeling, such as miR-132, which regulates BDNF expression. Its upregulation after SE impairs synaptic plasticity and promotes epileptogenesis [149, 151]. MiR-204 increases apoptosis by inhibiting *Bcl-2* and Bak1; its downregulation in chronic epilepsy correlates with hippocampal neuron loss [152]. miR-128, whose pathogenic process is ion channel and neurotransmitter dysregulation, targets potassium channels and glutamate receptors, affecting neuronal excitability. His dysregulation is linked to seizure generalization [92, 153].

## miRNAs as Diagnostic Biomarkers

Circulating miRNAs in biofluids offer non-invasive diagnostic potential. And those miRNA samples can be taken from blood, serum, urine, and other fluids (Table 9). The first type is Blood-Based miRNA signatures. TLE-like plasma miR-106b-5p, miR-134-5p, and miR-122-5p are elevated in TLE patients versus controls (AUC: 0.86–0.91) [148, 155]. miR-155-5p distinguishes TLE from juvenile myoclonic epilepsy (JME), reflecting syndrome-specific dysregulation [155]. miR-27a-3p Pediatric Epilepsy is significantly downregulated in plasma (p < 0.0001) and correlates with disease severity [156]. A 158-miRNA signature (82 upregulated, 76 downregulated) differentiates epileptic children from controls [156]. miR-19b and miR-451 are enriched in CSF and reflect BBB disruption during seizures [148]. Their levels correlate with seizure frequency better than plasma miRNAs [92]. Multi-miRNA panels for enhanced accuracy, like a panel combining miR-21, miR-126, and miR-223, achieve 86% accuracy in distinguishing epilepsy from healthy controls [148, 152]. And finally, Urinary miR-320 + miR-203 detects early acute seizures with > 85% sensitivity [152].

**Table 9:** Clinically validated diagnostic miRNA panel

miRNA Panel	Biofluid	Epilepsy Type	Accuracy	Ref
miR-21, miR-126, miR-223	Plasma	General epilepsy	AUC: 0.86	[148]
miR-192	Serum	Diabetic epilepsy	AUC: 0.91	[155]
miR-320, miR-203	Urine	Acute seizures	Sens: 85%	[152]
miR-19b, miR-451	CSF	Refractory TLE	r = 0.78*	[92]

AUC: area under the curve, CSF: cerebrospinal fluid, Sens: sensitivity

## Prognostic Applications of miRNAs

MiRNAs predict disease progression, therapeutic response, and comorbidities. miRNAs that are involved in seizure recurrence and severity are miR-212-3p and miR-132-3p; low plasma levels 48 h post-trauma predict seizure clusters (≥ 3 seizures/24h) in post-traumatic epilepsy (PTE) (AUC: 0.81; 95% specificity) [152]. miR-1290 downregulation in glioblastoma-associated epilepsy correlates with higher seizure burden and shorter survival [154]. Other miRNAs are involved in drug resistance, such as miR-199a-3p, whose overexpression in TLE reduces P-glycoprotein expression, enhancing drug permeability. While low levels predict resistance to carbamazepine [148, 156]. miR-27a-3p suppresses *GOLM1* and *LIMK1* genes that are linked to drug efflux. Its downregulation correlates with resistance to levetiracetam in children [156]. Moreover, miRNAs predicting surgical outcomes like miR-134, whose high preoperative levels associate with post-surgical seizure freedom in TLE (p = 0.002) [92, 148].

## Limitations of miRNA Biomarkers in Epilepsy

miRNAs hold substantial promise as non-invasive biomarkers for neurological disorders; however, their clinical translation remains hindered by methodological variability and limited reproducibility [157]. Differences in sampling protocols, normalization strategies, and reference gene selection, combined with confounding factors such as age, sex, treatment history, and comorbidities, contribute to inconsistent expression patterns across studies. These technical and biological disparities underscore the absence of standardized analytical workflows continue to hinder the validation of reliable clinical biomarkers [158].

In epilepsy, several circulating miRNAs, including hsa-miR-134, miR-181a, and miR-146a, have been proposed as diagnostic candidates; however, their expression patterns are highly variable and often context dependent [158]. For instance, miR-134 shows decreased plasma levels in some patient groups but increased hippocampal expression associated with neuronal hyperexcitability and seizure susceptibility [73].

Similarly, miR-181a demonstrates a dynamic stage-specific profile downregulated during acute seizures, unchanged in the latent phase, and upregulated in chronic epilepsy, complicating its consistent diagnostic interpretation. Dysregulation of miR-146a, a modulator of the NF-κB inflammatory pathway, may yield either neuroprotective or neurotoxic outcomes depending on disease stage and cellular context [159]. Additional candidates such as miR-124, miR-199a, miR-128, miR-15a-5p, and miR-194-5p have shown potential diagnostic relevance, yet their reproducibility and specificity remain unconfirmed in large, multicenter cohorts [92, 150]. The heterogeneity of epilepsy syndromes, particularly between focal, generalized, and drug-resistant forms, further complicates the identification of universally applicable miRNA signatures [92].

Beyond epilepsy, miR-134 exemplifies the broader challenges of context-dependent biomarker interpretation across neurodegenerative and neuropsychiatric conditions. While it plays a pivotal role in dendritic spine formation, synaptic plasticity, and neuronal excitability, its direction of regulation varies markedly across diseases: it is upregulated in epilepsy but downregulated in major depressive disorder (MDD) and Alzheimer’s disease, reflecting differential modulation of the CREB–BDNF signaling axis [160, 161].

### Technical Considerations for miRNA Detection

The clinical translation of miRNAs as diagnostic and prognostic biomarkers critically depends on the accuracy, sensitivity, reproducibility, and inter-laboratory comparability of detection methodologies. Although multiple analytical platforms have been successfully employed for miRNA profiling, the absence of standardized workflows across pre-analytical, analytical, and post-analytical stages remains a major obstacle to their routine clinical implementation. Consequently, technical variability—rather than biological differences—often accounts for inconsistent miRNA signatures reported across studies [148, 152].

Pre-analytical variables constitute the dominant source of variability in miRNA detection and therefore require rigorous standardization to ensure analytical reliability and biological validity. Key factors include biological matrix selection (plasma, serum, cerebrospinal fluid, urine, or tissue), anticoagulant choice, processing time, centrifugation conditions, RNA extraction methodology, storage temperature, and freeze–thaw cycles. Hemolysis represents a critical confounder in circulating miRNA analyses, as erythrocyte-enriched miRNAs such as miR-16 and miR-451 can markedly distort expression profiles; thus, routine hemolysis assessment using spectrophotometric indices or miRNA ratio–based quality controls is strongly recommended [162]. To minimize inter-study variability and enhance reproducibility, the implementation of standardized operating procedures (SOPs) for sample collection, RNA isolation, spike-in controls, and RNA input normalization is essential, with transparent methodological reporting to enable cross-study comparability.

Analytical and post-analytical standardization is equally critical for transforming miRNA detection into a clinically actionable and reproducible tool. Quantitative reverse transcription PCR (qRT-PCR) remains the most widely applied platform due to its sensitivity, yet it is highly dependent on primer design, reverse transcription efficiency, and normalization strategy, necessitating strict adherence to MIQE guidelines and the use of validated multi-reference or global mean normalization approaches [163]. Next-generation sequencing (NGS) enables unbiased miRNA discovery and isomiR detection but is susceptible to library preparation bias and adapter ligation artifacts, underscoring the need for harmonized library protocols and transparent bioinformatic pipelines [164]. Digital PCR (ddPCR) provides absolute miRNA quantification with superior precision and tolerance to PCR inhibitors, making it particularly suitable for clinical validation and longitudinal monitoring [165]. Across all platforms, external quality controls, cross-platform harmonization, and standardized reporting frameworks are indispensable to ensure that observed miRNA signatures reflect true biological variation rather than technical artifacts.

## Conclusion

The exploration of miRNAs in epilepsy has unveiled their multifaceted roles in disease initiation and progression. By modulating gene expression, miRNAs influence neuronal excitability, synaptic architecture, and inflammatory responses, all of which are critical in the development of epileptic networks. The dysregulation of specific miRNAs can lead to aberrant neuronal firing and network synchronization, hallmark features of epilepsy. Understanding the precise mechanisms by which miRNAs contribute to epileptogenesis offers promising avenues for therapeutic intervention. Targeting miRNAs to restore normal gene expression patterns holds potential for developing novel ASMs. Additionally, the stability and detectability of miRNAs in bodily fluids position them as attractive candidates for non-invasive biomarkers, aiding in early diagnosis, prognosis, and monitoring of therapeutic responses. Future research should focus on elucidating the complex regulatory networks involving miRNAs, identifying key miRNA targets, and developing safe and effective miRNA-based therapeutics. Integrating miRNA profiling into clinical practice could revolutionize the management of epilepsy, enabling personalized treatment strategies and improving patient outcomes.

## Data Availability

No datasets were generated or analysed during the current study.

## References

[1] Hamamoto O, Tirapelli DPdC, Lizarte Neto FS, Freitas-Lima P, Saggioro FP, Cirino MLdA et al (2020) Modulation of NMDA receptor by miR-219 in the amygdala and hippocampus of patients with mesial temporal lobe epilepsy. J Clin Neurosci 74:180–186 32111564 10.1016/j.jocn.2020.02.024

[2] Xu Y, Zhang L, Yan Y, Xiao W, Zou W, Luo Z et al (2025) MicroRNA-33 regulates the synaptic plasticity-related gene ARC in temporal lobe epilepsy. Neurosci Res 210:19–27 39214315 10.1016/j.neures.2024.08.003

[3] Aziz N (2023) Exploring novel therapeutic strategies for the treatment of epilepsy-associated neuroinflammation. Sfera 10:2526135

[4] Mansour RM, Rizk NI, Abdel Mageed SS, Alam-Eldein KM, Fahmy HA, Lutfy RH et al (2025) miRNA dysregulation in depression: unraveling the interplay between neuroplasticity, HPA axis dysfunction, and neuroinflammation. Metab Brain Dis 40(8):308 41191100 10.1007/s11011-025-01730-7

[5] Balosso S, Vezzani A, Ravizza T (2021) Emerging molecular mechanisms of neuroinflammation in seizure disorders. Inflammation and epilepsy: New vistas. Springer, pp 21–43

[6] Manna I, Fortunato F, De Benedittis S, Sammarra I, Bertoli G, Labate A et al (2022) Non-coding RNAs: new biomarkers and therapeutic targets for temporal lobe epilepsy. Int J Mol Sci 23(6):3063 35328484 10.3390/ijms23063063 PMC8954985

[7] Brennan GP, Garcia-Curran MM, Patterson KP, Luo R, Baram T (2021) Multiple disruptions of glial-neuronal networks in epileptogenesis that follows prolonged febrile seizures. Front Neurol 12:615802 33679583 10.3389/fneur.2021.615802 PMC7930821

[8] de Liyis BG, Tandy SG, Endira JF, Putri KA, Utami DK (2022) Psychiatry, neurosurgery, Anti-high mobility group box protein 1 monoclonal antibody downregulating P-glycoprotein as novel epilepsy therapeutics. Psychiatry Neurosurg 58(1):121 10.1186/s41983-022-00557-8 PMC9589779 36310854

[9] Alam-ElDein KM, Faraag AH, El-Yamany NA, Abdel Moneim AE, Abdelfattah MS, El-Khadragy MF et al (2026) Studying the potential ameliorative effect of biosynthesized selenium nanoparticles using epigallocatechin gallate against depression in rats. Front Pharmacol 16:1691567 10.3389/fphar.2025.1691567 PMC12835393 41608023

[10] Dantio CD, Fasoranti DO, Teng C, Li X (2025) Seizures in brain tumors: pathogenesis, risk factors and management. Int J Mol Med 55(5):82 40116082 10.3892/ijmm.2025.5523 PMC11964414

[11] Sabet Sarvestani F, Azarpira N (2022) microRNAs alterations of myocardium and brain ischemia-reperfusion injury: insight to improve infarction. Immunol Investig 51(1):51–72 33028103 10.1080/08820139.2020.1808672

[12] Doghish AS, Rizk NI, Mohammed OA, Hemdan M, Alam‐Eldein KM, Basiouny MS et al (2026) MicroRNAs as key regulators and potential biomarkers in vitiligo pathogenesis. J Gene Med 28(1):e70083

[13] Villasana-Salazar B, Vezzani AJNoD (2023) Neuroinflammation microenvironment sharpens seizure circuit. Neurobiol Dis 178:106027 36736598 10.1016/j.nbd.2023.106027

[14] Kuzniewska B, Rejmak K, Nowacka A, Ziółkowska M, Milek J, Magnowska M et al (2022) Disrupting interaction between miR-132 and Mmp9 3′ UTR improves synaptic plasticity and memory in mice. Front Mol Neurosci 15:924534 35992198 10.3389/fnmol.2022.924534 PMC9389266

[15] Scărlătescu AI, Micheu MM, Popa-Fotea N-M, Dorobanțu MJ (2021) MicroRNAs in acute ST elevation myocardial infarction—a new tool for diagnosis and prognosis: therapeutic implications. Int J Mol Sci 22(9):4799 33946541 10.3390/ijms22094799 PMC8124280

[16] Eker ED, Korkulu R (2025) Identification of Novel miRNA Biomarkers for Epilepsy: a qRT-PCR-Based Profiling Study. Bratisl Med J 127:1–17

[17] Foiadelli T, Santangelo A, Costagliola G, Costa E, Scacciati M, Riva A et al (2023) Neuroinflammation and status epilepticus: a narrative review unraveling a complex interplay. Front Pediatr 11:1251914 38078329 10.3389/fped.2023.1251914 PMC10703175

[18] Sun C, Ren X, Wu F, Chen W, Wang J, Deng L (2024) Total Flavonoids of Eucommia ulmoides Oliver Protects Cardiomyocytes against Lipopolysaccharide-Induced Injury by Regulating microRNA-494 Expression. Indian J Pharm Sci 86(2):494

[19] Shariff S, Nouh HA, Inshutiyimana S, Kachouh C, Abdelwahab MM, Nazir A et al (2024) Advances in understanding the pathogenesis of epilepsy: unraveling the molecular mechanisms. Health Sci Rep 7(2):e1896 38361811 10.1002/hsr2.1896 PMC10867297

[20] Staroverov S, Kozlov S, Fomin A, Gabalov K, Khanadeev V, Soldatov D et al (2021) Synthesis of silymarin−selenium nanoparticle conjugate and examination of its biological activity in vitro. ADMET and DMPK 9.4:255–266 10.5599/admet.1023 PMC8920099 35300372

[21] Staley K (2015) Molecular mechanisms of epilepsy. Nat Neurosci 18(3):367–372 25710839 10.1038/nn.3947 PMC4409128

[22] Alyu F, Dikmen M (2017) Inflammatory aspects of epileptogenesis: contribution of molecular inflammatory mechanisms. Acta Neuropsychiatr 29(1):1–16 27692004 10.1017/neu.2016.47

[23] Lutfy RH, Ashour AM, Khames A, Elhemiely AA, Alam-ElDein KM, Faraag AHI et al (2025) Targeting oxidative stress and neuroinflammation: Epigallocatechin-3-gallate-Selenium nanoparticles mitigate sleep deprivation-induced cortical impairment. Int J Mol Sci. 10.3390/ijms262211173 41303666 10.3390/ijms262211173 PMC12653894

[24] Chugh D, Ali I, Bakochi A, Bahonjic E, Etholm L, Ekdahl CT (2015) Alterations in brain inflammation, synaptic proteins, and adult hippocampal neurogenesis during epileptogenesis in mice lacking Synapsin2. PLoS One 10(7):e0132366 26177381 10.1371/journal.pone.0132366 PMC4503715

[25] Michalak Z, Lebrun A, Miceli MD, Rousset M-C, Crespel A, Coubes P et al (2012) IgG leakage may contribute to neuronal dysfunction in drug-refractory epilepsies with blood-brain barrier disruption. J Neuropathol Exp Neurol 71(9):826–838 22878666 10.1097/NEN.0b013e31826809a6

[26] Rana A, Musto AE (2018) The role of inflammation in the development of epilepsy. J Neuroinflammation 15(1):144 29764485 10.1186/s12974-018-1192-7 PMC5952578

[27] Mukhtar I (2020) Inflammatory and immune mechanisms underlying epileptogenesis and epilepsy: from pathogenesis to treatment target. Seizure 82:65–79 33011590 10.1016/j.seizure.2020.09.015

[28] Marchi N, Granata T, Ghosh C, Janigro D (2012) Blood-brain barrier dysfunction and epilepsy: pathophysiologic role and therapeutic approaches. Epilepsia 53(11):1877–1886 22905812 10.1111/j.1528-1167.2012.03637.x PMC4842020

[29] Rojas A, Jiang J, Ganesh T, Yang MS, Lelutiu N, Gueorguieva P et al (2014) Cyclooxygenase-2 in epilepsy. Epilepsia 55(1):17–25 24446952 10.1111/epi.12461 PMC3956447

[30] Chen L, Zhu L, Lu D, Dai S, Han Y, Wu Z et al (2021) Association between autoimmune encephalitis and epilepsy: systematic review and meta-analysis. Seizure 91:346–359 34284303 10.1016/j.seizure.2021.07.005

[31] Miller DS, Bauer B, Hartz AM (2008) Modulation of P-glycoprotein at the blood-brain barrier: opportunities to improve central nervous system pharmacotherapy. Pharmacol Rev 60(2):196–209 18560012 10.1124/pr.107.07109 PMC2634288

[32] Sumadewi KT, Harkitasari S, Tjandra DC (2023) Biomolecular mechanisms of epileptic seizures and epilepsy: a review. Acta Epileptol 5(1):28 40217521 10.1186/s42494-023-00137-0 PMC11960269

[33] Lignani G, Baldelli P, Marra V (2020) Homeostatic plasticity in epilepsy. Front Cell Neurosci 14:197 32676011 10.3389/fncel.2020.00197 PMC7333442

[34] Bielefeld P, Mooney C, Henshall DC, Fitzsimons CP (2017) miRNA-mediated regulation of adult hippocampal neurogenesis; implications for epilepsy. Brain Plast 3(1):43–59 29765859 10.3233/BPL-160036 PMC5928558

[35] Dogini DB, Avansini SH, Vieira AS, Lopes-Cendes I (2013) MicroRNA regulation and dysregulation in epilepsy. Front Cell Neurosci 7:172 24109432 10.3389/fncel.2013.00172 PMC3790106

[36] Szydlowska K, Bot A, Nizinska K, Olszewski M, Lukasiuk K (2024) Circulating microRNAs from plasma as preclinical biomarkers of epileptogenesis and epilepsy. Sci Rep 14(1):708 38184716 10.1038/s41598-024-51357-4 PMC10771472

[37] Yakovleva KD, Dmitrenko DV, Panina IS, Usoltseva AA, Gazenkampf KA, Konovalenko OV et al (2022) Expression profile of miRs in mesial temporal lobe epilepsy: systematic review. Int J Mol Sci. 10.3390/ijms23020951 35055144 10.3390/ijms23020951 PMC8781102

[38] Zhang X, Ma Y, Zhou F, Zhang M, Zhao D, Wang X et al (2022) Identification of miRNA-mRNA regulatory network associated with the glutamatergic system in post-traumatic epilepsy rats. Front Neurol 13:1102672 36619916 10.3389/fneur.2022.1102672 PMC9822725

[39] Lanza M, Cuzzocrea S, Oddo S, Esposito E, Casili G (2023) The role of miR-128 in neurodegenerative diseases. Int J Mol Sci. 10.3390/ijms24076024 37046996 10.3390/ijms24076024 PMC10093830

[40] Israni DK, Patel ML, Dodiya RK (2024) Exploring the versatility of miRNA-128: a comprehensive review on its role as a biomarker and therapeutic target in clinical pathways. Mol Biol Rep 51(1):860 39068606 10.1007/s11033-024-09822-w

[41] Bhattacharyya P, Biswas A, Biswas SC (2022) Brain-enriched miR-128: reduced in exosomes from Parkinson’s patient plasma, improves synaptic integrity, and prevents 6-OHDA mediated neuronal apoptosis. Front Cell Neurosci 16:1037903 36713778 10.3389/fncel.2022.1037903 PMC9879011

[42] Bhattacharyya P, Biswas A, Biswas SC (2022) Brain-enriched miR-128: reduced in exosomes from Parkinson’s patient plasma, improves synaptic integrity, and prevents 6-OHDA mediated neuronal apoptosis. Front Cell Neurosci 16:1037903 10.3389/fncel.2022.1037903 PMC9879011 36713778

[43] Wang C, Dong M, Zhang X, Wang X, Zhao Y, Cao Y (2023) Competitive binding of circCCDC6 to microRNA-128-3p activates TXNIP/NLRP3 pathway and promotes cerebral ischemia-reperfusion defects. Acta Biochim Pol 70(4):807–815 37934513 10.18388/abp.2020_6552

[44] Tan CL, Plotkin JL, Veno MT, von Schimmelmann M, Feinberg P, Mann S et al (2013) MicroRNA-128 governs neuronal excitability and motor behavior in mice. Science 342(6163):1254–1258 24311694 10.1126/science.1244193 PMC3932786

[45] Brodie MJ (2010) Antiepileptic drug therapy the story so far. Seizure 19(10):650–655 21075011 10.1016/j.seizure.2010.10.027

[46] Reschke CR, Henshall DC (2015) microRNA and epilepsy. microRNA: sedical Evidence: from molecular biology to clinical practice. Adv Exp Med Biol 888:41–70 10.1007/978-3-319-22671-2_4 26663178

[47] Schratt GM, Tuebing F, Nigh EA, Kane CG, Sabatini ME, Kiebler M et al (2006) A brain-specific microRNA regulates dendritic spine development. Nature 439(7074):283–289 16421561 10.1038/nature04367

[48] Jimenez-Mateos EM, Engel T, Merino-Serrais P, Fernaud-Espinosa I, Rodriguez-Alvarez N, Reynolds J et al (2015) Antagomirs targeting microRNA-134 increase hippocampal pyramidal neuron spine volume in vivo and protect against pilocarpine-induced status epilepticus. Brain Struct Funct 220(4):2387–2399 24874920 10.1007/s00429-014-0798-5

[49] Wang XM, Jia RH, Wei D, Cui WY, Jiang W (2014) MiR-134 blockade prevents status epilepticus like-activity and is neuroprotective in cultured hippocampal neurons. Neurosci Lett 572:20–25 24810882 10.1016/j.neulet.2014.04.049

[50] Kim YJ, Kim SH, Park Y, Park J, Lee JH, Kim BC (2020) miR-16-5p is upregulated by amyloid beta deposition in Alzheimer’s disease models and induces neuronal cell apoptosis through direct targeting and suppression of BCL-2. Exp Gerontol 136:110954 32320719 10.1016/j.exger.2020.110954

[51] Zhang R, Zhou H, Jiang L, Mao Y, Cui X, Xie B et al (2016) MiR-195 dependent roles of mitofusin2 in the mitochondrial dysfunction of hippocampal neurons in SAMP8 mice. Brain Res 1652:135–143 27693395 10.1016/j.brainres.2016.09.047

[52] Lungu G, Stoica G, Ambrus A (2013) MicroRNA profiling and the role of microRNA-132 in neurodegeneration using a rat model. Neurosci Lett 553:153–158 23973300 10.1016/j.neulet.2013.08.001

[53] Chaudhuri AD, Choi DC, Kabaria S, Tran A, Junn E (2016) MicroRNA-7 regulates the function of mitochondrial permeability transition pore by targeting VDAC1 expression. J Biol Chem 291(12):6483–6493 26801612 10.1074/jbc.M115.691352 PMC4813563

[54] Gowda P, Reddy PH, Kumar S (2022) Deregulated mitochondrial microRNAs in Alzheimer’s disease: focus on synapse and mitochondria. Ageing Res Rev 73:101529 34813976 10.1016/j.arr.2021.101529 PMC8692431

[55] Korotkov A, Broekaart DW, Banchaewa L, Pustjens B, van Scheppingen J, Anink JJ et al (2020) microRNA-132 is overexpressed in glia in temporal lobe epilepsy and reduces the expression of pro-epileptogenic factors in human cultured astrocytes. Glia 68(1):60–75 31408236 10.1002/glia.23700 PMC6899748

[56] Jimenez-Mateos EM, Bray I, Sanz-Rodriguez A, Engel T, McKiernan RC, Mouri G et al (2011) miRNA Expression profile after status epilepticus and hippocampal neuroprotection by targeting miR-132. Am J Pathol 179(5):2519–2532 21945804 10.1016/j.ajpath.2011.07.036 PMC3204080

[57] Brennan GP, Dey D, Chen Y, Patterson KP, Magnetta EJ, Hall AM et al (2016) Dual and opposing roles of microRNA-124 in epilepsy are mediated through inflammatory and NRSF-dependent gene networks. Cell Rep 14(10):2402–2412 26947066 10.1016/j.celrep.2016.02.042 PMC4794429

[58] Ma Y (2018) The challenge of microRNA as a biomarker of epilepsy. Curr Neuropharmacol 16(1):37–42 28676013 10.2174/1570159X15666170703102410 PMC5771381

[59] Lin ST, Huang Y, Zhang L, Heng MY, Ptacek LJ, Fu YH (2013) MicroRNA-23a promotes myelination in the central nervous system. Proc Natl Acad Sci U S A 110(43):17468–17473 24101522 10.1073/pnas.1317182110 PMC3808585

[60] Song YJ, Tian XB, Zhang S, Zhang YX, Li X, Li D et al (2011) Temporal lobe epilepsy induces differential expression of hippocampal miRNAs including let-7e and miR-23a/b. Brain Res 1387:134–140 21376023 10.1016/j.brainres.2011.02.073

[61] Sun C, Zhu L, Ma R, Ren J, Wang J, Gao S et al (2019) Astrocytic miR-324-5p is essential for synaptic formation by suppressing the secretion of CCL5 from astrocytes. Cell Death Dis 10(2):141 30760705 10.1038/s41419-019-1329-3 PMC6374376

[62] Stappert L, Borghese L, Roese-Koerner B, Weinhold S, Koch P, Terstegge S et al (2013) MicroRNA-based promotion of human neuronal differentiation and subtype specification. PLoS ONE 8(3):e59011 23527072 10.1371/journal.pone.0059011 PMC3601127

[63] Jerng HH, Pfaffinger PJ, Covarrubias M (2004) Molecular physiology and modulation of somatodendritic A-type potassium channels. Mol Cell Neurosci 27(4):343–369 15555915 10.1016/j.mcn.2004.06.011

[64] Moshe SL, Perucca E, Ryvlin P, Tomson T (2015) Epilepsy: new advances. Lancet 385(9971):884–898 25260236 10.1016/S0140-6736(14)60456-6

[65] Schaefer N, Roemer V, Janzen D, Villmann C (2018) Impaired glycine receptor trafficking in neurological diseases. Front Mol Neurosci 11:291 30186111 10.3389/fnmol.2018.00291 PMC6110938

[66] Lee ST, Jeon D, Chu K, Jung KH, Moon J, Sunwoo J et al (2017) Inhibition of miR-203 reduces spontaneous recurrent seizures in mice. Mol Neurobiol 54(5):3300–3308 27165289 10.1007/s12035-016-9901-7

[67] Fan C, Wu Q, Ye X, Luo H, Yan D, Xiong Y et al (2016) Role of miR-211 in neuronal differentiation and viability: Implications to pathogenesis of Alzheimer’s Disease. Front Aging Neurosci 8:166 27458373 10.3389/fnagi.2016.00166 PMC4937029

[68] Bekenstein U, Mishra N, Milikovsky DZ, Hanin G, Zelig D, Sheintuch L et al (2017) Dynamic changes in murine forebrain miR-211 expression associate with cholinergic imbalances and epileptiform activity. Proc Natl Acad Sci U S A 114(25):E4996–E5005 28584127 10.1073/pnas.1701201114 PMC5488936

[69] Webster KM, Sun M, Crack P, O’Brien TJ, Shultz SR, Semple BD (2017) Inflammation in epileptogenesis after traumatic brain injury. J Neuroinflamm 14(1):10 10.1186/s12974-016-0786-1 PMC5237206 28086980

[70] Feng Y, Yang H, Yue Y, Tian F (2020) MicroRNAs and target genes in epileptogenesis. Epilepsia 61(10):2086–2096 32944964 10.1111/epi.16687

[71] Rocchi A, Moretti D, Lignani G, Colombo E, Scholz-Starke J, Baldelli P et al (2019) Neurite-enriched MicroRNA-218 stimulates translation of the GluA2 subunit and increases excitatory synaptic strength. Mol Neurobiol 56(8):5701–5714 30671783 10.1007/s12035-019-1492-7

[72] Sonawane S, Vsiansky V, Brazdil M (2024) MicroRNA-mediated regulation of neurotransmitter receptors in epilepsy: a systematic review. Epilepsy Behav 158:109912 38924965 10.1016/j.yebeh.2024.109912

[73] Jimenez-Mateos EM, Engel T, Merino-Serrais P, McKiernan RC, Tanaka K, Mouri G et al (2012) Silencing microRNA-134 produces neuroprotective and prolonged seizure-suppressive effects. Nat Med 18(7):1087–1094 22683779 10.1038/nm.2834 PMC3438344

[74] Reschke CR, Henshall DC (2015) MicroRNA and epilepsy. Adv Exp Med Biol 888:41–70 26663178 10.1007/978-3-319-22671-2_4

[75] Lambert TJ, Storm DR, Sullivan JM (2010) MicroRNA132 modulates short-term synaptic plasticity but not basal release probability in hippocampal neurons. PLoS ONE 5(12):e15182 21206919 10.1371/journal.pone.0015182 PMC3012071

[76] Wingo TS, Yang J, Fan W, Min Canon S, Gerasimov ES, Lori A et al (2020) Brain microRNAs associated with late-life depressive symptoms are also associated with cognitive trajectory and dementia. NPJ Genom Med 5:6 32047652 10.1038/s41525-019-0113-8 PMC7004995

[77] Bicker S, Khudayberdiev S, Weiss K, Zocher K, Baumeister S, Schratt G (2013) The DEAH-box helicase DHX36 mediates dendritic localization of the neuronal precursor-microRNA-134. Genes Dev 27(9):991–996 23651854 10.1101/gad.211243.112 PMC3656329

[78] Sun J, Gao X, Meng D, Xu Y, Wang X, Gu X et al (2017) Antagomirs targeting MicroRNA-134 increase Limk1 levels after experimental seizures in vitro and in vivo. Cell Physiol Biochem 43(2):636–643 28942448 10.1159/000480647

[79] Gao J, Wang WY, Mao YW, Graff J, Guan JS, Pan L et al (2010) A novel pathway regulates memory and plasticity via SIRT1 and miR-134. Nature 466(7310):1105–1109 20622856 10.1038/nature09271 PMC2928875

[80] Fiore R, Rajman M, Schwale C, Bicker S, Antoniou A, Bruehl C et al (2014) MiR-134-dependent regulation of Pumilio-2 is necessary for homeostatic synaptic depression. EMBO J 33(19):2231–2246 25097251 10.15252/embj.201487921 PMC4282509

[81] Napoli D, Pizzorusso T (2017) miRNA in neuronal networks maturation and plasticity. Academic Press 12:211–224

[82] Magill ST, Cambronne XA, Luikart BW, Lioy DT, Leighton BH, Westbrook GL et al (2010) MicroRNA-132 regulates dendritic growth and arborization of newborn neurons in the adult hippocampus. Proc Natl Acad Sci U S A 107(47):20382–20387 21059906 10.1073/pnas.1015691107 PMC2996687

[83] Wayman GA, Davare M, Ando H, Fortin D, Varlamova O, Cheng HY et al (2008) An activity-regulated microRNA controls dendritic plasticity by down-regulating p250GAP. Proc Natl Acad Sci U S A 105(26):9093–9098 18577589 10.1073/pnas.0803072105 PMC2449370

[84] Kannan M, Lee SJ, Schwedhelm-Domeyer N, Nakazawa T, Stegmuller J (2012) P250GAP is a novel player in the Cdh1-APC/Smurf1 pathway of axon growth regulation. PLoS ONE 7(11):e50735 23226367 10.1371/journal.pone.0050735 PMC3511349

[85] Fu N, Yu J, Zhu L, Yang L, Ma L, He J et al (2023) Role of miR-219a-5p in regulating NMDAR in nonylphenol-induced synaptic plasticity damage. Ecotoxicol Environ Saf 252:114576 36736231 10.1016/j.ecoenv.2023.114576

[86] Dugas JC, Cuellar TL, Scholze A, Ason B, Ibrahim A, Emery B et al (2010) Dicer1 and miR-219 are required for normal oligodendrocyte differentiation and myelination. Neuron 65(5):597–611 20223197 10.1016/j.neuron.2010.01.027 PMC2843397

[87] Zheng H, Tang R, Yao Y, Ji Z, Cao Y, Liu Z et al (2016) MiR-219 protects against seizure in the Kainic Acid model of epilepsy. Mol Neurobiol 53(1):1–7 25394384 10.1007/s12035-014-8981-5

[88] Kocerha J, Faghihi MA, Lopez-Toledano MA, Huang J, Ramsey AJ, Caron MG et al (2009) MicroRNA-219 modulates NMDA receptor-mediated neurobehavioral dysfunction. Proc Natl Acad Sci U S A 106(9):3507–3512 19196972 10.1073/pnas.0805854106 PMC2651305

[89] Alsharafi WA, Xiao B, Li J (2016) MicroRNA-139-5p negatively regulates NR2A-containing NMDA receptor in the rat pilocarpine model and patients with temporal lobe epilepsy. Epilepsia 57(11):1931–1940 27731509 10.1111/epi.13568

[90] Wang L, Song L, Chen X, Suo J, Ma Y, Shi J et al (2020) MicroRNA-139-5p confers sensitivity to antiepileptic drugs in refractory epilepsy by inhibition of MRP1. CNS Neurosci Ther 26(4):465–474 31750616 10.1111/cns.13268 PMC7080432

[91] Jimenez-Mateos E, Henshall D (2013) Epilepsy and microRNA. Neuroscience 238:218–229 23485811 10.1016/j.neuroscience.2013.02.027

[92] Jeelani M (2024) miRNAs in epilepsy: a review from molecular signatures to therapeutic intervention. Int J Biol Macromol 263:130468 38417757 10.1016/j.ijbiomac.2024.130468

[93] Mao S, Wu J, Yan J, Zhang W, Zhu F (2023) Dysregulation of miR-146a: a causative factor in epilepsy pathogenesis, diagnosis, and prognosis. Front Neurol 14:1094709 37213914 10.3389/fneur.2023.1094709 PMC10196196

[94] Aronica E, Fluiter K, Iyer A, Zurolo E, Vreijling J, Van Vliet E et al (2010) Expression pattern of miR‐146a, an inflammation‐associated microRNA, in experimental and human temporal lobe epilepsy. Eur J Neurosci 31(6):1100–1107 20214679 10.1111/j.1460-9568.2010.07122.x

[95] Alzahrani S, Al Doghaither H, Al‑ghafari A, Pushparaj P (2023) 5‑Fluorouracil and capecitabine therapies for the treatment of colorectal cancer (review). Oncol Rep 50(4):175 37594133 10.3892/or.2023.8612

[96] Basu T, Maguire J, Salpekar JA (2021) Hypothalamic-pituitary-adrenal axis targets for the treatment of epilepsy. Neurosci Lett 746:135618 33429002 10.1016/j.neulet.2020.135618

[97] Dwivedi Y, Roy B, Lugli G, Rizavi H, Zhang H, Smalheiser N (2015) Chronic corticosterone-mediated dysregulation of microRNA network in prefrontal cortex of rats: relevance to depression pathophysiology. Transl Psychiatry 5(11):e682–e682 26575223 10.1038/tp.2015.175 PMC5068767

[98] Haramati S, Navon I, Issler O, Ezra-Nevo G, Gil S, Zwang R et al (2011) MicroRNA as repressors of stress-induced anxiety: the case of amygdalar miR-34. J Neurosci 31(40):14191–14203 21976504 10.1523/JNEUROSCI.1673-11.2011 PMC6623664

[99] Zacharjasz J, Sztachera M, Smuszkiewicz M, Piwecka M (2024) Micromanaging the neuroendocrine system–a review on miR-7 and the other physiologically relevant miRNAs in the hypothalamic–pituitary axis. FEBS Lett 598(13):1557–1575 38858179 10.1002/1873-3468.14948

[100] Brennan GP, Henshall DC (2018) microRNAs in the pathophysiology of epilepsy. Neurosci Lett 667:47–52 28104433 10.1016/j.neulet.2017.01.017

[101] Kobow K, Blümcke I (2018) Epigenetics in epilepsy. Neurosci Lett 667:40–46 28111355 10.1016/j.neulet.2017.01.012

[102] Aronica E, Gorter JA (2007) Gene expression profile in temporal lobe epilepsy. Neuroscientist 13(2):100–108 17404370 10.1177/1073858406295832

[103] Zhang C, Wang C, Chen X, Yang C, Li K, Wang J et al (2010) Expression profile of microRNAs in serum: a fingerprint for esophageal squamous cell carcinoma. Clin Chem 56(12):1871–1879 20943850 10.1373/clinchem.2010.147553

[104] Iori V, Maroso M, Rizzi M, Iyer AM, Vertemara R, Carli M et al (2013) Receptor for Advanced Glycation Endproducts is upregulated in temporal lobe epilepsy and contributes to experimental seizures. Neurobiol Dis 58:102–114 23523633 10.1016/j.nbd.2013.03.006

[105] Vezzani A, Aronica E, Mazarati A, Pittman QJ (2013) Epilepsy and brain inflammation. Exp Neurol 244:11–21 21985866 10.1016/j.expneurol.2011.09.033

[106] Wang H, Ye Y, Zhu Z, Mo L, Lin C, Wang Q et al (2016) MiR-124 regulates apoptosis and autophagy process in MPTP model of P arkinson’s disease by targeting to B im. Brain Pathol 26(2):167–176 25976060 10.1111/bpa.12267 PMC8029438

[107] Stevanovic M, Ninkovic DS, Mojsin M, Drakulic D, Schwirtlich M (2022) Interplay of SOX transcription factors and microRNAs in the brain under physiological and pathological conditions. Neural Regen Res 17(11):2325–2334 35535866 10.4103/1673-5374.338990 PMC9120710

[108] Vezzani A, French J, Bartfai T, Baram TZ (2011) The role of inflammation in epilepsy. Nat Rev Neurol 7(1):31–40 21135885 10.1038/nrneurol.2010.178 PMC3378051

[109] Ma L, Semick SA, Chen Q, Li C, Tao R, Price AJ et al (2020) Schizophrenia risk variants influence multiple classes of transcripts of sorting nexin 19 (SNX19). Mol Psychiat 25(4):831–843 10.1038/s41380-018-0293-0 30635639

[110] Yousefi MJ, Rezvanimehr A, Saleki K, Mehrani A, Barootchi E, Ramezankhah M et al (2025) Inflammation-related microRNA alterations in epilepsy: a systematic review of human and animal studies. Rev Neurosci 8:901–923 10.1515/revneuro-2025-0041 40755381

[111] Mostafa DK, Gaber DE, Hassaan PS, Dwedar FI, Dief AE, Fathelbab MHJTEJoN et al (2025) Crosstalk between miRNAs, high mobility group box-1 and complement system in epilepsy: relevance of response to valproic acid. Egypt J Neurol Psychiatry Neurosurg 61(1):81

[112] Lovisari F (2020) microRNAs, gene networks, cell therapy: promises and challenges for treating epilepsies and their comorbidities. Sfera 10:2478815 10.1016/j.yebeh.2019.106488 31494060

[113] Tiwari D, Peariso K, Gross C (2018) MicroRNA-induced silencing in epilepsy: opportunities and challenges for clinical application. Dev Dyn 247(1):94–110 28850760 10.1002/dvdy.24582 PMC5740004

[114] Pal S, Mandal S (2025) Therapeutic potential of microRNAs in neurological disorders: mechanisms, biomarkers, and emerging therapeutic strategies. Explor Neuroprotect Ther 5:1004111

[115] Srivastava A, Dixit AB, Banerjee J, Tripathi M, Chandra PS (2016) Role of inflammation and its miRNA based regulation in epilepsy: implications for therapy. Clin Chim Acta 452:1–9 26506013 10.1016/j.cca.2015.10.023

[116] Reschke CR, Silva LFA, Norwood BA, Senthilkumar K, Morris G, Sanz-Rodriguez A et al (2017) Potent anti-seizure effects of locked nucleic acid antagomirs targeting miR-134 in multiple mouse and rat models of epilepsy. Mol Therap Nucleic acids 6:45–56 28325299 10.1016/j.omtn.2016.11.002 PMC5363384

[117] Wang D, Li Z, Zhang Y, Wang G, Wei M, Hu Y et al (2016) Targeting of micro RNA-199a-5p protects against pilocarpine-induced status epilepticus and seizure damage via SIRT 1–p53 cascade. Epilepsia 57(5):706–716 26945677 10.1111/epi.13348

[118] Tiwari D, Rajathi V, Rymer JK, Beasley LN, McGann AM, Bunk AT et al (2023) Estradiol-and progesterone-associated changes in microRNA-induced silencing and reduced antiseizure efficacy of an antagomir in female mice. eNeuro. 10.1523/eneuro.0047-22.2023 37433683 10.1523/ENEURO.0047-22.2023 PMC10368146

[119] Gross C, Yao X, Engel T, Tiwari D, Xing L, Rowley S et al (2016) MicroRNA-mediated downregulation of the potassium channel Kv4. 2 contributes to seizure onset. Cell Rep 17(1):37–45 27681419 10.1016/j.celrep.2016.08.074 PMC5061042

[120] Yuan J, Huang H, Zhou X, Liu X, Ou S, Xu T et al (2016) MicroRNA-132 interact with p250GAP/Cdc42 pathway in the hippocampal neuronal culture model of acquired epilepsy and associated with epileptogenesis process. Neural Plast 2016(1):5108489 27579184 10.1155/2016/5108489 PMC4992765

[121] Zhang H, Lian Y, Xie N, Cheng X, Chen C, Xu H et al (2020) Antagomirs targeting miR-142–5p attenuate pilocarpine-induced status epilepticus in mice. Exp Cell Res 393(2):112089 32439493 10.1016/j.yexcr.2020.112089

[122] Yi TT, Zhang LM, Huang XN (2023) Glycyrrhizic acid protects against temporal lobe epilepsy in young rats by regulating neuronal ferroptosis through the miR-194-5p/PTGS2 axis. Kaohsiung J Med Sci 39(2):154–165 36647717 10.1002/kjm2.12642 PMC11895926

[123] Ma Z, Dang HA, Yang J, Rodella G, Mwema A, De Lombaerde E et al (2026) Lipid nanoparticle-mediated delivery of microRNA-124 reduces neuroinflammation. Biomaterials 325:123589 40753784 10.1016/j.biomaterials.2025.123589

[124] Reschke CR, Silva LFA, Vangoor VR, Rosso M, David B, Cavanagh BL et al (2021) Systemic delivery of antagomirs during blood-brain barrier disruption is disease-modifying in experimental epilepsy. Mol Ther 29(6):2041–2052 33609732 10.1016/j.ymthe.2021.02.021 PMC8178478

[125] De Matteis M, Cecchetto G, Munari G, Balsamo L, Gardiman MP, Giordano R et al (2018) Circulating miRNAs expression profiling in drug-resistant epilepsy: up-regulation of miR-301a-3p in a case of sudden unexpected death. Leg Med Tokyo 31:7–9 29220722 10.1016/j.legalmed.2017.12.003

[126] Wong JC (2024) MicroRNA 335-5p: the sodium channel silencer. Epilepsy Curr 24(1):50–52 38327537 10.1177/15357597231212373 PMC10846517

[127] Xiang L, Ren Y, Li X, Zhao W, Song Y (2016) MicroRNA-204 suppresses epileptiform discharges through regulating TrkB-ERK1/2-CREB signaling in cultured hippocampal neurons. Brain Res 1639:99–107 26947618 10.1016/j.brainres.2016.02.045

[128] Zheng H, Tang R, Yao Y, Ji Z, Cao Y, Liu Z et al (2016) MiR-219 protects against seizure in the kainic acid model of epilepsy. Mol Neurobiol 53:1–7 25394384 10.1007/s12035-014-8981-5

[129] Tao H, Zhao J, Liu T, Cai Y, Zhou X, Xing H et al (2017) Intranasal delivery of miR‐146a mimics delayed seizure onset in the lithium‐pilocarpine mouse model. Mediators Inflamm 2017(1):6512620 28242958 10.1155/2017/6512620 PMC5294386

[130] Di-mi ZH, Lu GA, Lin CH (2023) Cheng-fang, Effect of inhibiting the expression of miRNA-193a-5p on hippocampal neuron protection in epileptic model rats, Chinese J Contemp Neurol Neurosurg 23(3):223

[131] Jiang Y, Li L, Tan X, Liu B, Zhang Y, Li C (2015) miR-210 mediates vagus nerve stimulation-induced antioxidant stress and anti-apoptosis reactions following cerebral ischemia/reperfusion injury in rats. J Neurochem 134(1):173–181 25783636 10.1111/jnc.13097

[132] Zhao Q, Yin C, Yuan Y, Zhang H, Teng L (2019) Down-regulation of Mir-145 improves learning and memory abilities in epileptic rats by regulating hippocampal neuron apoptosis. World Neurosurg 122:e1432–e1438 30465949 10.1016/j.wneu.2018.11.080

[133] Wang W, Guo Y, He L, Chen C, Luo J, Ma Y et al (2018) Overexpression of miRNA-137 in the brain suppresses seizure activity and neuronal excitability: a new potential therapeutic strategy for epilepsy. Neuropharmacology 138:170–181 29894770 10.1016/j.neuropharm.2018.06.010

[134] Gerbatin RR, Augusto J, Morris G, Campbell A, Worm J, Langa E et al., (2022) Investigation of MicroRNA-134 as a Target against Seizures and SUDEP in a Mouse Model of Dravet Syndrome. eNeuro 9(5):1–9 10.1523/ENEURO.0112-22.2022 PMC9522462 36240080

[135] Brennan GP, Henshall DC (2020) MicroRNAs as regulators of brain function and targets for treatment of epilepsy. Nat Rev Neurol 16(9):506–519 32546757 10.1038/s41582-020-0369-8

[136] Shyam M, Bm O, Srirangan PNN, Sabina EP (2025) Targeted miRNA delivery in epilepsy: mechanisms, advances, and therapeutic potential. Mol Biol Rep 52(1):1–31 10.1007/s11033-025-10436-z 40192852

[137] Rzepka-Migut B, Kruszniewska-Rajs C, Bugowska M, Krzempek M, Gola J, Striano P et al (2025) Circulating microRNAs as potential biomarkers in pediatric epilepsy: a pilot study. Epilepsy Behav 171:110631 40752237 10.1016/j.yebeh.2025.110631

[138] Wang Y, Chen C, Ya C, Chen J, Lu B, Liu J, Wu Q, Diao L et al (2025) The levels of miR-155 in epilepsy patients: a meta-analysis. Front Neurol 16:1630581 40881793 10.3389/fneur.2025.1630581 PMC12380531

[139] Zakaria F, Yousef SA, AbdelDayem J, ElGamal R, Issa OY, Mansour M et al (2025) The Role of MicroRNAs in the Pathogenesis and as Biomarkers for Pediatric Epilepsy: A Systematic Review 29(5):571 10.1007/s40291-025-00791-9 PMC12436486 40484870

[140] Morris G, O’Brien D, Henshall DC (2021) Opportunities and challenges for microRNA-targeting therapeutics for epilepsy. Trends Pharmacol Sci 42(7):605–616 33992468 10.1016/j.tips.2021.04.007

[141] Kasaiyan M, Basiri M, Pajouhanfar S (2025) The role of miRNA134 in pathogenesis and treatment of intractable epilepsy: a review article. Nucleosides Nucleotides Nucleic Acids 44(3):222–237 38531025 10.1080/15257770.2024.2331046

[142] Morris G, Reschke CR, Henshall DC (2019) Targeting microRNA-134 for seizure control and disease modification in epilepsy. EBioMedicine 45:646–654 31300345 10.1016/j.ebiom.2019.07.008 PMC6642437

[143] Toonen LJA, Casaca-Carreira J, Pellisé-Tintoré M, Mei H, Temel Y, Jahanshahi A et al (2018) Intracerebroventricular administration of a 2’-O-Methyl Phosphorothioate Antisense Oligonucleotide results in activation of the innate immune system in Mouse brain. Nucleic Acid Ther 28(2):63–73 29565739 10.1089/nat.2017.0705 PMC5899290

[144] McCartan R, Khorkova O, Volmar CH, Wahlestedt C (2023) Nucleic acid-based therapeutics for the treatment of central nervous system disorders. Front Genet 14:1250276 37662844 10.3389/fgene.2023.1250276 PMC10468602

[145] Brillante S, Volpe M, Indrieri A (2024) Advances in MicroRNA therapeutics: from preclinical to clinical studies. Hum Gene Ther 35(17–18):628–648 39150011 10.1089/hum.2024.113

[146] Diener C, Keller A, Meese E (2022) Emerging concepts of miRNA therapeutics: from cells to clinic. Trends Genet 38(6):613–626 35303998 10.1016/j.tig.2022.02.006

[147] Mageed SSA, Rashad AA, Elshaer SS, Elballal MS, Mohammed OA, Darwish SF et al (2024) The emerging role of miRNAs in epilepsy: from molecular signatures to diagnostic potential. Pathol Res Pract 254:155146 38266457 10.1016/j.prp.2024.155146

[148] Guarnieri L, Amodio N, Bosco F, Carpi S, Tallarico M, Gallelli L et al (2024) Circulating miRNAs as novel clinical biomarkers in temporal lobe epilepsy. Non-coding RNA 10(2):18 38525737 10.3390/ncrna10020018 PMC10961783

[149] Wang J, Zhao J (2021) MicroRNA dysregulation in epilepsy: from pathogenetic involvement to diagnostic biomarker and therapeutic agent development. Front Mol Neurosci 14:650372 33776649 10.3389/fnmol.2021.650372 PMC7994516

[150] Wang J, Zhao J (2021) MicroRNA dysregulation in epilepsy: from pathogenetic involvement to diagnostic biomarker and therapeutic agent development. Front Mol Neurosci 14:650372 33776649 10.3389/fnmol.2021.650372 PMC7994516

[151] Alsharafi WA, Xiao B, Abuhamed MM, Luo Z (2015) miRNAs: biological and clinical determinants in epilepsy. Front Mol Neurosci 8:59 26528124 10.3389/fnmol.2015.00059 PMC4602137

[152] Heiskanen M, Ndode-Ekane XE, Ali I, Santana-Gomez C, Puhakka N, Gupta SD et al (2025) Plasma microRNAs as prognostic biomarkers for development of severe epilepsy after experimental traumatic brain injury—EpiBioS4Rx Project 1 study. Epilepsia 66(3):870–885 39661396 10.1111/epi.18219 PMC11908664

[153] Cui S, Yu S, Huang H-Y, Lin Y-C-D, Huang Y, Zhang B et al (2025) miRTarBase 2025: updates to the collection of experimentally validated microRNA–target interactions. Nucleic Acids Res 53(D1):D147–D156 39578692 10.1093/nar/gkae1072 PMC11701613

[154] Zhang Z, Li Q, Fan X, You G (2025) Significance of miR-1290 in glioblastoma patients with epilepsy. Sci Rep 15(1):13911 40263483 10.1038/s41598-025-97855-x PMC12015410

[155] Timechko EE, Lysova KD, Yakimov AM, Paramonova AI, Vasilieva AA, Kantimirova EA et al (2025) Circulating microRNAs as biomarkers of various forms of epilepsy. Med Sci Basel 13(1):7 39846702 10.3390/medsci13010007 PMC11755555

[156] Wang Q, Shi X, Li P-P, Gao L, Zhou Y, Li L et al (2024) microRNA profilings identify plasma biomarkers and targets associated with pediatric epilepsy patients. Pediatr Res 95(4):996–1008 37884644 10.1038/s41390-023-02864-z PMC10920196

[157] Brennan GP, Henshall DC (2020) MicroRNAs as regulators of brain function and targets for treatment of epilepsy. Nat Rev Neurol 16(9):506–519 32546757 10.1038/s41582-020-0369-8

[158] Zhang HP, Liu XL, Chen JJ, Cheng K, Bai SJ, Zheng P et al (2020) Circulating microRNA 134 sheds light on the diagnosis of major depressive disorder. Transl Psychiatry 10(1):95 32179735 10.1038/s41398-020-0773-2 PMC7075934

[159] Ma Y (2018) The challenge of microRNA as a biomarker of epilepsy. Curr Neuropharmacol 16(1):37–42 28676013 10.2174/1570159X15666170703102410 PMC5771381

[160] Ding R, Su D, Zhao Q, Wang Y, Wang J-Y, Lv S et al (2023) The role of micrornas in depression. Front Pharmacol 14:1129186 37063278 10.3389/fphar.2023.1129186 PMC10090555

[161] Musazzi L, Mingardi J, Ieraci A, Barbon A, Popoli M (2023) Stress, microRNAs, and stress-related psychiatric disorders: an overview. Mol Psychiatry 28(12):4977–4994 37391530 10.1038/s41380-023-02139-3

[162] Blondal T, Nielsen SJ, Baker A, Andreasen D, Mouritzen P, Teilum MW et al (2013) Assessing sample and miRNA profile quality in serum and plasma or other biofluids. Methods 59(1):S1–S6 23036329 10.1016/j.ymeth.2012.09.015

[163] Bustin SA, Benes V, Garson JA, Hellemans J, Huggett J, Kubista M et al (2009) The MIQE guidelines: minimum information for publication of quantitative real-time PCR experiments. Clin Chem 55(4):611–622 19246619 10.1373/clinchem.2008.112797

[164] Hafner M, Renwick N, Brown M, Mihailović A, Holoch D, Lin C et al (2011) RNA-ligase-dependent biases in miRNA representation in deep-sequenced small RNA cDNA libraries. RNA 17(9):1697–1712 21775473 10.1261/rna.2799511 PMC3162335

[165] Hindson BJ, Ness KD, Masquelier DA, Belgrader P, Heredia NJ, Makarewicz AJ et al (2011) High-throughput droplet digital PCR system for absolute quantitation of DNA copy number. Anal Chem 83(22):8604–8610 22035192 10.1021/ac202028g PMC3216358

